# The impact of primary health care on AIDS incidence and mortality: A cohort study of 3.4 million Brazilians

**DOI:** 10.1371/journal.pmed.1004302

**Published:** 2024-07-11

**Authors:** Priscila F. P. S. Pinto, James Macinko, Andréa F. Silva, Iracema Lua, Gabriela Jesus, Laio Magno, Carlos A. S. Teles Santos, Maria Yury Ichihara, Mauricio L. Barreto, Corrina Moucheraud, Luis E. Souza, Inês Dourado, Davide Rasella

**Affiliations:** 1 Institute of Collective Health, Federal University of Bahia (ISC/UFBA), Salvador, Brazil; 2 The Centre for Data and Knowledge Integration for Health (CIDACS-Fiocruz), Salvador, Brazil; 3 Departments of Health Policy and Management and Community Health Sciences, Fielding School of Public Health, University of California (UCLA), Los Angeles, California, United States of America; 4 Department of Life Sciences, State University of Bahia (UNEB), Salvador, Brazil; 5 Instituto de Salud Global Barcelona (ISGlobal), Hospital Clínic—Universitat de Barcelona, Barcelona, Spain; Washington University in St Louis School of Medicine, UNITED STATES OF AMERICA

## Abstract

**Background:**

Primary Health Care (PHC) is essential for effective, efficient, and more equitable health systems for all people, including those living with HIV/AIDS. This study evaluated the impact of the exposure to one of the largest community-based PHC programs in the world, the Brazilian Family Health Strategy (FHS), on AIDS incidence and mortality.

**Methods and findings:**

A retrospective cohort study carried out in Brazil from January 1, 2007 to December 31, 2015. We conducted an impact evaluation using a cohort of 3,435,068 ≥13 years low-income individuals who were members of the 100 Million Brazilians Cohort, linked to AIDS diagnoses and deaths registries. We evaluated the impact of FHS on AIDS incidence and mortality and compared outcomes between residents of municipalities with low or no FHS coverage (unexposed) with those in municipalities with 100% FHS coverage (exposed). We used multivariable Poisson regressions adjusted for all relevant municipal and individual-level demographic, socioeconomic, and contextual variables, and weighted with inverse probability of treatment weighting (IPTW). We also estimated the FHS impact by sex and age and performed a wide range of sensitivity and triangulation analyses; 100% FHS coverage was associated with lower AIDS incidence (rate ratio [RR]: 0.76, 95% CI: 0.68 to 0.84) and mortality (RR: 0.68, 95%CI: 0.56 to 0.82). FHS impact was similar between men and women, but was larger in people aged ≥35 years old both for incidence (RR: 0.62, 95% CI: 0.53 to 0.72) and mortality (RR: 0.56, 95% CI: 0.43 to 0.72). The absence of important confounding variables (e.g., sexual behavior) is a key limitation of this study.

**Conclusions:**

AIDS should be an avoidable outcome for most people living with HIV today and our study shows that FHS coverage could significantly reduce AIDS incidence and mortality among low-income populations in Brazil. Universal access to comprehensive healthcare through community-based PHC programs should be promoted to achieve the Sustainable Development Goals of ending AIDS by 2030.

## Abstract 2

Priscila Pinto and colleagues assess the impact of a community-based primary health program—the Brazilian Family Health Strategy—on HIV/AIDS incidence and HIV-related mortality in Brazil.

## Author summary

**Why was this study done?:**

- The Family Health Strategy (FHS), the main model for Primary Health Care (PHC) in Brazil, has been associated with improved healthcare access and quality as well as better health outcomes.
- Although PHC is identified as a key element to achieve the goal of ending AIDS by 2030, the impact of FHS on HIV/AIDS outcomes has not been studied.

**What did the researchers do and find?:**

- Using a large cohort of 3.4 million low-income Brazilians, we evaluate the impact of FHS on AIDS incidence and mortality.
- We found lower AIDS incidence and mortality among individuals exposed to FHS throughout the study period.

**What do these findings mean?:**

- FHS is a robust community-based PHC model associated with improved population health outcomes including for people living with HIV/AIDS.
- While promising, these findings must be interpreted cautiously considering that some confounders, such as sexual behavior, were unavailable.

## Introduction

In 2021, about 38.4 million people were living with HIV (PLWH) globally, with 650,000 people dying from AIDS-related illnesses [1]. Strengthening Primary Health Care (PHC) has been identified as a key strategy to achieve the goal of ending AIDS by 2030 [2]. PHC is an integrated set of health services that, when well-designed, and with universal and equitable coverage, provide comprehensive and coordinated care, including prevention and treatment for people living with HIV/AIDS (PLWHA) [3,4]. HIV prevention takes place in PHC settings through voluntary counseling and testing, health promotion, and the prescribing of antivirals and pre- and post-exposure prophylaxis (PrEP and PEP) for non–HIV-infected patients [4]. Community-based PHC can promote the use of condoms and safer-sex behaviors, increase HIV testing cost-effectively, enabling early diagnosis, access and adherence to antiretroviral therapy (ART) resulting in reductions in HIV transmission, increasing ART uptake, leading to better viral suppression and retention in care, lower incidence and lower mortality in low- and middle-income countries (LMICs) contexts, reducing HIV/AIDS coinfections, comorbidities, and deaths [5–11].

Moreover, decentralizing services to a countrywide PHC network, that is, redistributing care for people with HIV/AIDS from specialized health services to PHC should enhance recognition of population needs and improve testing uptake and linkage to care among key populations such as men who have sex with men, female sex workers, and injecting drug users who have historically had difficulty accessing services through conventional healthcare providers [12].

The Family Health Strategy (FHS) is the way Brazil’s national health system provides community-based PHC and is one of the world’s largest and most evaluated nationwide PHC strategies. FHS characteristics include: universal access with no payment for services; coordinated and comprehensive preventive and curative care delivered by a multidisciplinary team of a physician, a nurse, and community health workers (CHW); geographic empanelment of about 3,500 people residing near the health center’s location; and monthly visits by a CHW who often lives in the area [13]. The FHS carries out rapid tests for HIV in the community, and updated clinical protocols have encouraged monitoring people with HIV at this level of care [13]. While the FHS has been associated with improved healthcare access and quality as well as better health outcomes [14], its impact on HIV/AIDS outcomes has never been studied to our knowledge [15]. Thus, the aim of this study was to evaluate the impact of the 100% coverage of a nationwide community-based PHC strategy on AIDS incidence and mortality using a unique cohort of 3.4 million lower-income Brazilians. Our hypothesis is that, among those with HIV, FHS exposure reduces the risk for developing or dying from AIDS.

## Methods

### Study design, population, and ethical issues

In this cohort study, the population was based on longitudinal data from a cohort of 3.4 million low-income individuals aged 13 and older followed up for 9 years. These individuals represent a subgroup of the “100 Million Brazilians Cohort.” The study protocol was previously published [16].

This study was approved by the research ethics committee of the Federal University of Bahia, under number 41691315.0.0000.5030 (Report No: 3.783.920). The “100 Million Brazilians Cohort” is based on the linkage of administrative data and did not require an additional consent form.

## Data sources

The “100 Million Brazilians Cohort” was previously constructed using the Brazilian national database for social benefits—the Cadastro Único (CadUnico)—which comprises the poorest half of the Brazilian population and is used in determining eligibility for social welfare programs [17]. This cohort was then linked to: (1) information from all nationwide HIV/AIDS confirmed diagnoses (National Disease Notification System [SINAN]); and (2) and deaths records and their causes classified by International Classification of Diseases (ICD-10) codes including nationwide confirmed deaths from HIV/AIDS (Mortality Information System [SIM]) (see S1 Appendix p.2). These databases were combined using the previously validated CIDACS-Record Linkage tool (https://gitHub.com/gcgbarbosa/cidacs-rl) based on the name of the individual and that of their mother, date of birth, sex, and municipality of residence in a two-step procedure [17,18]. In the first step, the linkage is deterministic. In the second step, entries that were not linked deterministically were then linked probabilistically based on a similarity score between all the pairwise comparisons (i.e., ranging from 0 to 1, where 1 means that is perfectly similar); entries with the highest similarity scores (above 0.95) were considered to be linked pairs [18,19]. The quality of each link for all causes between CadUnico, SINAN e SIM has been extensively evaluated and validated [17]. A database from the Ministry of Health with municipal FHS coverage (https://egestorab.saude.gov.br/) was deterministically linked to CadUnico records through the municipal code present in both databases. Annual FHS coverage was calculated according to the Brazilian Ministry of Health definition: the total number of FHS teams deployed in the municipality in that year multiplied by 3,450—representing the average number of individuals served by each FHS team—divided by the population of the municipality (S1 Appendix p. 2 to 3).

**Fig 1:**
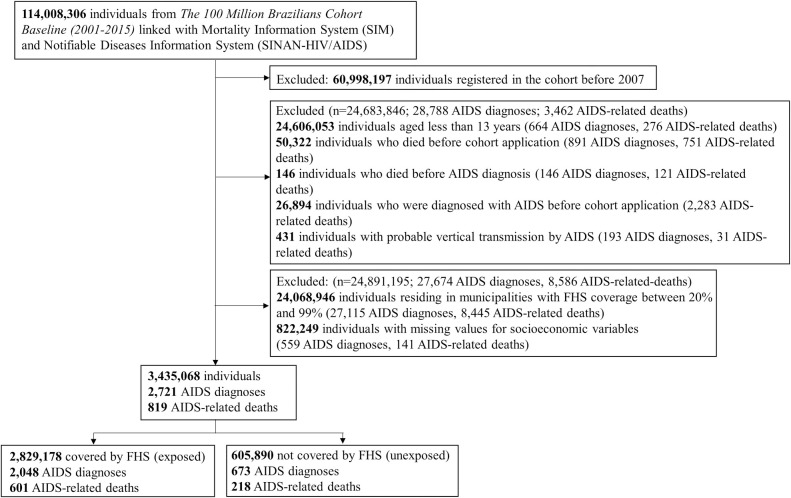
Selection flowchart of the study cohort, The 100 Million Brazilians Cohort, 2007–15.

The “100 Million Brazilians Cohort” contains information on 114,008,306 individuals between January 1, 2001 and December 31, 2015. To develop our analytical sample we excluded: (1) all people registered in the cohort before January 1, 2007 and with inconsistent dates; (2) all individuals infected with HIV through vertical transmission; (3) all individuals resident in municipalities with FHS coverage between 20% and 99% throughout the study period; (4) all individuals without complete information for all covariates (Fig 1). It is important to highlight that to deal with missing data, we carried out an analysis of complete cases due to the computational limitations of carrying out multiple imputation in large and complex databases such as the one used in the study.

The exposed group was composed of individuals residing in municipalities with 100% FHS coverage throughout the study period. We used a threshold of <20% FHS coverage for the comparison group—instead of lower coverage levels of 0% to 10%—as described in the study protocol [16]—in order to: (1) capture a higher number of individuals (considering the very low number of municipalities with zero [63] or up to 10% FHS coverage [71] over the all study period); (2) given the evidence from the literature that low FHS coverage is largely ineffective [20]; (3) because these thresholds has been used by similar individual-based PHC evaluation studies [21]; and (4) because FHS needs a municipal coverage greater than 70% to realize its potential according to previous studies [20]. However, lower thresholds have been tested in the sensitivity analyses.

## Outcomes

Outcomes included: (1) AIDS incidence (number of new AIDS cases divided by person-years *100,000); new AIDS cases are defined by adapted Centers for Disease Control and Prevention (CDC) criteria, Rio de Janeiro/Caracas criteria, and Death Criterion [22]; and (2) AIDS mortality rate (number of deaths from AIDS divided by person-years *100,000); AIDS deaths where the underlying cause of death was ICD-10 codes B20 to B24 [22]. We calculated the rate difference by subtracting the incidence rate in the unexposed group from the incidence rate in the exposed group, we also replicate this calculation for mortality. Follow-up time was calculated from the date of entry into the cohort until diagnosis of AIDS, death from AIDS, death from any cause, or the end of the cohort (December 31, 2015), whichever came first. Therefore, possible competing events (i.e., non-AIDS-related mortality) were censored.

## Statistical analyses

We performed descriptive analyses of the study population by FHS coverage for each outcome and for selected independent variables. These included demographic characteristics (sex and age), socioeconomic status (skin color, per capita household expenditures, educational attainment, home construction material, number of individuals per room), year of entry into the cohort, time of receipt of the Brazilian conditional cash transfer program Bolsa Família (in months, 0 if the family was not a beneficiary), and annual cumulative AIDS incidence for each individual’s municipality of residence from the study cohort, used as proxy of the municipal-level endemic burden of AIDS.

We selected these variables based on a conceptual framework of the main structural determinants of HIV infection, AIDS incidence and mortality, and of the hypothesized impact of PHC on each step of the evolution of the disease (S1 Appendix, p. 5). Associations between 100% FHS coverage (exposure) and AIDS incidence and mortality rates were estimated using multivariable Poisson regression models adjusted for all these relevant individual and municipal-level demographic and socioeconomic confounding variables, including follow-up time as an offset variable, with observations weighted using stabilized truncated inverse probability of treatment weighting (IPTW). The IPTW Poisson regression model is a method to evaluate the impact of PHC, and other interventions, with the 100 Million Brazilians Cohort [21]. Moreover, IPTW allows the construction of a control group suitable for impact assessment studies. Thus, we could compare a group exposed to FHS with those who did not.

We first calculated the propensity score (PS) using multivariable logistic regression models that take into account the probability that each individual lives in a municipality with 100% FHS coverage. Independent variables of the multivariable logistic regression included all the baseline demographic and socioeconomic characteristics cited above. Multivariable logistic regression models for the prediction of the propensity scores, and the odds ratios (OR) for each covariate are presented in S1 Appendix, p. 8. Considering Pt as the marginal probability of treatment (Pt, i.e., exposure to FHS in this study) in the population and PSmul as the propensity score obtained from the multivariable logistic regression adjusted for all independent variables, we calculated the IPTW using the formula (1 –Pt)/(1 –Psmul) for unexposed individuals and used the formula Pt/Psmul for exposed individuals. The extreme weights were truncated, i.e., we set allowable thresholds: a maximum (99th percentile) and a minimum (1st percentile) threshold. Therefore, individuals with a weight greater than 99th percentile and individuals with a weight lower than 1st percentile were assigned the allowable threshold instead of their actual weight. We then adjusted the IPT weighted multivariable Poisson regression using the same demographic and socioeconomic covariates used to calculate the propensity score along with: household economic conditions (water supply, type of lighting, garbage disposal, and sewage), and municipal conditions (unemployment rate, hospital beds per 1,000 inhabitants). For each model, the robust variance estimator was used and multicollinearity was assessed using variance inflation factors (VIF). Subgroup analyses included models by sex and age (under or over the mean age, i.e., 13 to 34 years and ≥35 years), and additional stratifications were used as complementary analysis (S1 Appendix, p. 10).

## Sensitivity analyses

Sensitivity analyses (S1 Appendix, p. 11–20) included first, investigating the impact of defining the comparison group as those living in municipalities with either 0% or with 10% FHS coverage over the study period, to test if the threshold used for the unexposed individuals (20%) affected our estimates. We also include analysis of data from municipalities across the spectrum of FHS coverage levels. Second, we performed a negative binomial regression model comparing results to those found using the IPTW Poisson regression model to evaluate the robustness of results to other specifications. Third, we performed the same multivariable Poisson regressions without IPTW to check for potential bias without weighting. Fourth, we conducted IPTW regressions without the municipal covariates to identify whether FHS coverage was truly driving the observed association or whether it was simply collinear with other municipal characteristics. Fifth, we fitted the IPTW Poisson regressions only with a subset of municipalities with high quality of vital information—according to consolidated criteria [23]—to check if the results are different from the main analysis. Sixth, mortality from external causes (ICD-10 codes V01-Y98) was included as a negative control, since the FHS was not expected to influence them. Seventh, marital status was included in the IPTW regressions as adjusting variable—even with its high number of missing values—to verify if it was a relevant confounder. Eighth, to assess the differences between exposed and unexposed individuals in relation to sexual preferences and behavior, we estimated the percentage of MSM—a variable only available in the records of AIDS cases—in the 2 groups. We also conducted complementary tests dividing the analysis in 2 periods, i.e., up to 2010 and after 2011, to check possible differences related to changes in treatments and protocols over the period. We chose 2011 because in this year PHC started carrying out rapid HIV tests [12] (S1 Appendix, p. 21–28). We also used the records of HIV cases from 2014, the year in which they started to be compulsorily notified, to assess if FHS exposure was also associated with potential HIV reductions (even if available study period was of only 2 years). Ninth, we performed a descriptive analysis about the differences in outcomes between the included and non-included due missing information (S1 Appendix, p. 7–8). Tenth, we performed the Poisson IPTW regression analysis account for clustering within the municipality (S1 Appendix, p. 12). Finally, we performed a triangulation test using an alternative methodology [24] with Cox regression (survival analysis) to estimate the FHS impact on AIDS incidence and compared these findings with our main results (S1 Appendix, p. 28). All analyses were performed using STATA version 15.0.

## Results

We studied 3,435,068 individuals—605,890 lived in municipalities with zero (or very low) FHS coverage and 2,829,178 lived in municipalities with full FHS coverage during the entire study period (Fig 1). As presented in Table 1, the main differences between the groups were: compared to unexposed municipalities, exposed municipalities had more men (44.7% versus 55.6%), people of pardo skin color (38.0% versus 63.9%), lower percentage of people with per capita expenses greater than 1 minimum monthly wage (20.7% versus 7.3%), less individuals with more than 9 years of education (32.2% versus 22.5%), with public network for garbage disposal (94.5% versus 63.4%) and for sewage (78.0% versus 25.0%).

**Table 1:** Individuals exposed and unexposed by the Family Health Strategy, The 100 Million Brazilians Cohort, Brazil, 2007–15.

	Unexposed *n* = 605,890	Exposed *n* = 2,829,178
	Rate (95% CI)	Rate (95% CI)
Incidence/100,000 persons-years at risk [1]	25.57 (23.71–27.58)	13.21 (12.65–13.80
Mortality/100,000 persons-years at risk [2]	8.28 (7.25–9.45)	3.88 (3.58–4.20)
	***N* (%)**	***N* (%)**
Sex		
Female	335,123 (55.3)	1,313,027 (46.4)
Male	270,767 (44.7)	1,516,151 (53.6)
Age (years)		
13–24	164,746 (27.2)	936,715 (33.1)
25–59	353,953 (58.4)	1,489,187 (52.6)
≥60	87,191 (14.4)	403,276 (14.3)
Skin color		
White	330,124 (54.5)	811,384 (28.7)
Asian	2,252 (0.4)	12,737 (0.4)
Pardo	230,255 (38.0)	1,807,417 (63.9)
Black	42,775 (7.0)	184,566 (6.5)
Indigenous	484 (0.1)	13,074 (0.5)
Per capita household expenditures—% MW		
>1	125,660 (20.7)	206,429 (7.3)
0.5–1	172,715 (28.5)	519,210 (18.4)
0.25–0.49	78,011 (12.9)	402,676 (14.2)
0–0.24	119,368 (19.7)	623,509 (22.0)
Nothing declared	110,136 (18.2)	1,077,354 (38.1)
Education (years of study)		
>9	194,862 (32.2)	637,528 (22.5)
4–9	165,858 (27.4)	686,351 (24.3)
1–4	197,210 (32.5)	1,003,227 (35.5)
Illiterate, never attended school	47,960 (7.9)	502,072 (17.7)
Water supply		
Public network	535,461 (88.4)	1,814,420 (64.1)
Well, spring or cistern	70,429 (11.6)	1,014,758 (35.9)
Home construction material		
Brick	558,976 (92.3)	2,113,608 (74.7)
Wood or taipa [3]	46,914 (7.7)	715,570 (25.3)
Lighting		
Electricity	554,155 (91.5)	2,504,416 (88.5)
No electricity	51,735 (8.5)	324,762 (11.5)
Number of individuals per room		
1	570,153 (94.1)	2,723,088 (96.2)
1–2	31,326 (5.2)	92,767 (3.3)
>2	4,411 (0.7)	13,323 (0.5)
Garbage disposal		
Public network	572,471 (94.5)	1,794,901 (63.4)
Burned, buried or another	33,419 (5.5)	1,034,277 (36.6)
Sewage		
Public network	472,462 (78.0)	708,550 (25.0)
Septic tank	34,699 (5.7)	621,743 (22.0)
Rudimentary cesspit/ditch or another	98,729 (16.3)	1,498,885 (53.0)
Year of entry into the cohort		
2007	93,461 (15.4)	867,836 (30.7)
2008	45,980 (7.6)	284,819 (10.1)
2009	42,595 (7.0)	255,916 (9.0)
2010	56,117 (9.3)	213,462 (7.6)
2011	75,914 (12.5)	228,745 (8.1)
2012	90,888 (15.0)	339,253 (12.0)
2013	51,864 (8.6)	228,104 (8.1)
2014	82,339 (13.6)	252,160 (8.9)
2015	66,732 (11.0)	158,883 (5.6)
	Mean (SD)	Mean (SD)
Time receiving Bolsa Família, months	30.13 (39.70)	52.27 (54.14)
AIDS municipal incidence among individuals in the cohort [4]	112.45 (100.23)	70.09 (76.28)
Municipal unemployment rate, %	7.00 (3.08)	7.70 (4.17)
Hospital beds per 1,000 inhabitants	1.97 (1.56)	1.96 (1.99)

%MW–Proportional to the baseline minimum wage (MW). SD–standard deviation.

1- Person-years at risk for incidence: 2631808.4 for unexposed group and 155001881 for exposed group.

2- Person-years at risk for incidence: 2633128 for unexposed group and 15506126 for exposed group.

3- Taipa is a construction method that consists of using clay and wood to build houses.

4- Annual cumulative AIDS incidence for each individual’s municipality of residence from the study cohort.

There were 2,721 new cases of AIDS during the study period, the mean AIDS incidence rate over the period was higher (25.57 per 100,000 person-years, 95% CI 23.71 to 27.58) among individuals living in municipalities with no FHS coverage, than among individuals living in municipalities with 100% FHS coverage (13.21 per 100,000 person-years, 95% CI 12.65 to 13.80) resulting in a rate difference of 12,36/100,000 person-years. During follow-up, 819 AIDS deaths occurred, in the unexposed group the AIDS mortality rate was higher (8.28 per 100,000 person-years, 95% CI 7.25 to 9.45) than among the exposed group (3.88 per 100,000 person-years, 95% CI 3.58 to 4.20) resulting in a rate difference of 4,4/100,000 person-years (Table 1).

FHS coverage was associated with a reduction in AIDS incidence (RR: 0.76, 95% CI 0.68 to 0.84) and with a reduction in AIDS-related mortality (RR: 0.68, 95% CI 0.56 to 0.82) (Table 2). Demographic and socioeconomic variables associated with an increase in AIDS incidence and mortality were: being aged 25 to 59 years, pardo and black skin color, having a lower level of education, and, having a lower per capita expenditure, while being aged ≥60 and reside in a home whose garbage disposal was not in the public network were associated with a decrease in AIDS incidence (Table 2).

**Table 2:** Inverse probability of treatment weighting Poisson regression models, adjusted for all demographic and socioeconomic variables, for the association between AIDS incidence and mortality rate and Family Health Strategy coverage in the study cohort, Brazil, 2007–15.

Variables	AIDS incidence *n* = 3,435,068	AIDS mortality *n* = 3,435,068
	Rra (CI 95%)	Rra (CI 95%)
FHS coverage		
<20%	1	1
100%	0.76 (0.68–0.84)	0.68 (0.56–0.82)
Sex		
Female	1	1
Male	1.11 (1.02–1.21)	1.15 (0.98–1.34)
Age (years)		
13–24	1	1
25–59	1.97 (1.79–2.17)	3.52 (2.86–4.32)
≥60	0.40 (0.31–0.52)	0.95 (0.63–1.43)
Skin color		
White	1	1
Asian	1.43 (0.74–2.77)	1.73 (0.55–5.42)
Pardo	1.38 (1.25–1.52)	1.51 (1.25–1.82)
Black	1.72 (1.49–1.99)	1.83 (1.42–2.36)
Indigenous	1.08 (0.54–2.17)	0.51 (0.07–3.63)
Education (years of study)		
>9	1	1
4–9	1.52 (1.34–1.72)	2.41 (1.85–3.14)
1–4	1.42 (1.25–1.61)	2.32 (1.78–3.02)
No education	1.27 (1.08–1.48)	2.26 (1.65–3.11)
Per capita expenditures—% MW		
>1	1	1
0.5–1	1.45 (1.16–1.82)	1.86 (1.18–2.95)
0.25–0.49	1.79 (1.41–2.27)	1.99 (1.22–3.26)
0–0.24	2.00 (1.57–2.56)	2.67 (1.62–4.39)
Nothing declared	2.21 (1.71–2.83)	2.79 (1.67–4.63)
Home construction material		
Brick	1	1
Wood or taipa [1]	1.18 (1.07–1.31)	1.10 (0.91–1.33)
Water supply		
Public network	1	1
Well, spring or cistern	0.89 (0.80–1.00)	0.85 (0.69–1.04)
Lighting		
Electricity	1	1
No electricity	1.18 (1.04–1.34)	1.15 (0.91–1.46)
Garbage disposal		
Public network	1	1
Burned, buried or another	0.65 (0.57–0.73)	0.70 (0.56–0.89)
Sewage		
Public network	1	1
Septic tank	0.81 (0.71–0.91)	0.80 (0.64–1.01)
Rudimentary cesspit/ditch or another	0.90 (0.81–1.00)	0.89 (0.73–1.07)
Number of individuals per room		
1	1	1
1–2	1.02 (0.82–1.26)	1.28 (0.84–1.95)
>2	0.78 (0.47–1.31)	0.73 (0.27–2.01)
Year of entry into the cohort		
2007	1	1
2008	1.14 (1.01–1.29)	1.01 (0.81–1.26)
2009	1.03 (0.90–1.18)	1.02 (0.80–1.30)
2010	1.16 (1.00–1.34)	0.92 (0.70–1.20)
2011	1.12 (0.93–1.36)	1.02 (0.72–1.45)
2012	1.27 (1.05–1.53)	1.13 (0.81–1.58)
2013	1.40 (1.11–1.78)	1.09 (0.68–1.75)
2014	1.45 (1.11–1.88)	1.07 (0.63–1.81)
2015	0.98 (0.59–1.65)	0.18 (0.02–1.31)
AIDS municipal incidence among individuals in the cohort [2]	1.00 (1.00–1.00)	1.00 (1.00–1.00)
Time receiving Bolsa Família, months	1.00 (1.00–1.00)	1.00 (0.99–1.00)
Municipal unemployment rate	1.00 (0.99–1.01)	1.01 (0.98–1.03)
Hospital beds per 1,000 inhabitants	1.05 (1.03–1.07)	1.06 (1.03–1.09)

Rra = rate risk adjusted by sex, age, skin color, education, per capita expenditures, home construction material, number of people per room, year of entry into the cohort, time receiving Bolsa Família (in months), AIDS municipal incidence in the cohort, water supply, lighting, sewage, garbage disposal, municipal unemployment rate, hospital beds per 1,000 inhabitants.

1- Taipa is a construction method that consists of using clay and wood to build houses.

2- Annual cumulative AIDS incidence for each individual’s municipality of residence from the study cohort.

CI, confidence interval; FHS, Family Health Strategy; %MW, proportional to the baseline minimum wage.

In the subgroup analyses (Table 3), the FHS association with AIDS incidence was slightly larger among women (RR: 0.70, 95% CI 0.61 to 0.82), but was significantly stronger for people aged 35 and older (RR: 0.62, 95% CI 0.53 to 0.72). Regarding AIDS mortality, the FHS association was slightly larger among men (RR: 0.64, 95% CI 0.49 to 0.83) and stronger among people aged 35 and older (RR: 0.56, 95% CI 0.43 to 0.72) (Table 3).

**Table 3:** Inverse probability of treatment weighting Poisson regression models, adjusted for all demographic and socioeconomic variables, for the association between AIDS incidence and mortality and Family Health Strategy coverage by sex and age, Brazil, 2007–15.

Variables	AIDS Incidence	AIDS Mortality
	*n* = 3,435,068	*n* = 3,435,068
	**Rra (95% CI)**	**Rra (95% CI)**
Sex		
Female	0.70 (0.61–0.82)	0.71 (0.54–0.93)
Male	0.79 (0.69–0.91)	0.64 (0.49–0.83)
Age (years)		
13–34	0.83 (0.72–0.96)	0.77 (0.58–1.03)
≥35	0.62 (0.53–0.72)	0.56 (0.43–0.72)

RRa = rate risk adjusted for sex, age, skin color, education, per capita expenditures, home construction material, number of people per room, year of entry into the cohort, time receiving Bolsa Família (in months), AIDS municipal rate in the cohort, water supply, lighting, sewage, garbage disposal, municipal unemployment rate, and hospital beds per 1,000 inhabitants.

CI, confidence interval.

In the sensitivity analyses using all levels of FHS coverage, we found a gradient, i.e., the greater the FHS coverage, the greater the reduction in AIDS incidence and mortality (S1 Appendix, p. 11). The other sensitivity analyses also confirmed the robustness of the findings, and all triangulation analyses showed a high degree of confidence in the impact estimation (S1 Appendix, p. 11–28).

## Discussion

To the best of our knowledge, this is the first study that evaluates the impact of a nationwide, community-based PHC strategy on AIDS incidence and mortality in a low- and middle-income country. Using a cohort of 3,435,068 individuals and 2,721 new AIDS cases with a robust cohort design, we found a strong impact of FHS coverage on the reduction of both AIDS incidence and mortality. Moreover, FHS showed a stronger impact among individuals ≥35 years old. A key limitation of this investigation is that important confounding variables (e.g., sexual behavior) were not available in our study.

These findings add to evidence that the FHS is a robust community-based PHC model associated with improved population health outcomes [20,21]. Another recent study using a similar cohort and design demonstrated that the FHS was also negatively associated with tuberculosis morbidity and mortality [21].

Strengthening and expanding PHC are recommended strategies to end AIDS by 2030 [3]. The rationale is that PHC is highly accessible, especially for those with greater social vulnerability [2], who are heavily affected by AIDS incidence and mortality [25]. PHC can foster greater trust with patients and by serving as the entry point to the health system can better facilitate access to HIV diagnosis through rapid testing, linkage to treatment services and enable early initiation of ART, all of which are directly related to the reduction of AIDS incidence [2,26,27]. HIV prevention actions carried out in PHC, such as sex education, family planning with condom distribution, follow-up and counseling of pregnant and postpartum women living with HIV/AIDS, distribution of syringes and needles to reduce harm among injecting drug users and offering PrEP and PEP, can play an essential role in reducing the incidence of HIV and its progression to AIDS [3,28]. It is important to point out that PrEP and PEP were introduced in Brazil in 2018; therefore, its effects on the treatment were not evaluated in the present study [29].

PHC should be the ideal place for managing AIDS, since like other chronic conditions it requires maintaining long-term treatments [2] as well as dealing with other health problems unrelated to HIV, but that comes with the increased life expectancy of PLWH. Centralized services that focus only on HIV/AIDS services may increase stigma among people living with the disease [12] and are limited in their ability to provide comprehensive and integrated care. PHC services, like the FHS, establish long-term ties with users and serve as their medical home, which can increase adherence to AIDS treatment and reduce mortality [30]. In the FHS, regular home visits, carried out by CHWs, are an important tool for monitoring ART adherence, helping individuals to gain access to other health and social services, identifying other behavioral or environmental risk factors, and delivering tailored education interventions [30]. A previous study carried out in Brazil showed that the introduction of rapid testing combined with ART—regardless of the patient’s immunosuppression—reduced AIDS incidence and mortality by 60% and 73%, respectively [29].

The impacts of PHC on AIDS incidence and mortality were stronger among older people, a result that was also expected since older people in Brazil tend to have greater access to PHC services [31]. In Brazil, AIDS mortality is higher among the elderly [32], which makes the results of this study even more relevant. However, it is noteworthy that we also found a relevant impact of the FHS in reducing AIDS incidence among people aged 13 to 34 years. Due to their higher vulnerability to HIV/AIDS, younger populations are a national priority for preventive actions in Brazil [28]. Our findings are particularly relevant, given that there are studies showing that youths linked to care initiate ART and achieve viral suppression at lower rates than older adults due to their lower levels of interest in communicable and noncommunicable disease care (which is less prevalent among younger people) and competing priorities, such as those related to social, educational, and economic advancement, that outweighed younger adults’ health concerns, resulting in lower care engagement [33]. On the other hand, when adolescents and youth receive counseling regarding ART adherence, as well as support and referral for psychosocial problems, they had better retention in ART [34].

This study has limitations. First, the positive impacts of the FHS found in this study are not representative nationally, because all individuals included in the study were derived from the 100 Million Brazilians Cohort, which contains individuals of lower socioeconomic status. Even so, the AIDS incidence rate found in the total population of our study (15.0/100,000 inhabitants, 95% CI:14.4 to 15.6) was very similar to that of the country in 2021 (16.5/100,000 inhabitants) [22]. Furthermore, despite of the high quality and coverage of the Brazilian health information systems used, the administrative data are subject to underreporting and ill-defined error [35]. However, we tested the main analysis only among individuals residing in municipalities with high quality of vital information and we found similar results when compared with the main analysis (S1 Appendix, p. 10). Second, we estimated the impact of FHS on AIDS outcomes by adjusting models for multiple socioeconomic variables, nonetheless some unobserved confounders (such as specific characteristics of individuals or variations across municipalities) may not have been accounted for in propensity score-based models. For this reason, we incorporated a wide range of individual-level and municipal-level factors as covariates in regression analyses, and we included the endemic levels of AIDS in the municipality as an adjusting variable that represent non-observed factors associated with the local burden of AIDS before the implementation of the intervention. It is worth clarifying that the expansion and consolidation of the FHS as a public policy program in Brazil occurred in 2006. Since 1996 some municipalities, for which data is not available, already had free HIV/AIDS treatment services. We believe that the inclusion of municipal AIDS detection rates also allowed an adequate adjustment to the model to minimize possible disparities between these municipalities that already had well-established vertical services prior to the FHS. Results from sensitivity analyses showed that the inclusion of other variables related to inequalities, infrastructure, and healthcare assistance at the municipal level do not affect the FHS impact estimates (S1 Appendix, p. 12). Moreover, variables related to social support such as marital status and sexual behavior such as self-identification as men who have sex with men (MSM), which were not included in the main models because of the high number of missing values, were nevertheless equally distributed among exposed and unexposed groups and indicated a predominant heterosexual HIV transmission (self-declared MSM were 13.0% among all AIDS cases) in our cohort of low-income individuals, the percentage of MSM among the linked AIDS cases was particularly low compared to the national proportion of 42.9% in 2021 [21]. Furthermore, when we incorporated marital status in the regression models they did not change the direction or magnitude of the results (S1 Appendix, p. 15–18). Third, our outcome variables were AIDS-related incidence and mortality (and not HIV incidence) because mandatory notification for HIV only started in 2014 in Brazil [28]. However, complementary analyses showed a significant negative association between FHS coverage and HIV incidence in 2014 to 2015. Fourth, to avoid sparse data, we defined “unexposed” individuals as those living in municipalities with <20% FHS coverage. However, sensitivity analyses showed no relevant changes to our results when the unexposed groups were defined as those living in municipalities with 0% or <10% FHS coverage (S1 Appendix, p. 11). Fifth, to deal with missing data, we chose to exclude people who did not have complete information for the study’s independent variables from the study because of the computational limitations of carrying out multiple imputation in large and complex databases such as the one used in the study. This decision could not demonstrate the impact of the FHS among people with greater social vulnerability, who often do not have complete CadUnico registration. Nonetheless, the independent variables do not present a high number of missing information in the total study population (S1 Appendix, p.7–8). Finally, while the FHS has been found to be a robust model of PHC, not all FHS providers deliver care that is appropriate for some populations at high risk of HIV infection, such as LGBTQ+ individuals [36]. Enhancing the ability of PHC providers to make care more welcoming, appropriate and effective for key populations at risk for HIV infection remains a challenge for the FHS and for PHC in many country contexts.

The main strengths of our study are first its unprecedentedly large cohort of individuals, AIDS cases, and AIDS deaths accompanied by a wide range of sensitivity analyses—which confirmed the robustness of the findings—and triangulation tests—that indicate a high degree of confidence in the results.

In conclusion, our findings show that a universal model of community-based PHC could significantly reduce incidence and mortality from AIDS in LMICs and even decrease AIDS-related inequities among vulnerable populations. The results of this study justify the expansion and strengthening of effective PHC strategies globally to reach the Sustainable Development Goal of ending AIDS by 2030.

## Supporting information

S1 Appendix Supplemental methods and results. Detailed methodological description of the dataset, data sources, and conceptual framework of the study. Additionally, descriptive results by outcomes, results for logistic regression, complementary subgroup results, results for all sensitivity, and triangulation analyses.
(DOCX)

S1 STROBE Statement The RECORD statement—checklist of items, extended from the STROBE statement, that should be reported in observational studies using routinely collected health data. (DOCX)

S1 Fig Conceptual framework of selected structural determinants of HIV infection, AIDS incidence and mortality, and of the hypothesized effects of Primary Health Care and Family Health Strategy on this process. (TIF)

## Abbreviations

ART: antiretroviral therapy
CHW: community health worker
FHS: Family Health Strategy
IPTW: inverse probability of treatment weighting
LMIC: low- and middle-income country
MSM: men who have sex with men
OR: odds ratio
PEP: post-exposure prophylaxis
PHC: Primary Health Care
PLWH: people were living with HIV
PS: propensity score
RR: rate ratio
VIF: variance inflation factor
