# Supplementary material for: The impact of primary health care on AIDS incidence and mortality: A cohort study of 3.4 million Brazilians
Source: PLoS Med. 2024 Jul 11;21(7):e1004302. doi: 10.1371/journal.pmed.1004302 (PMC11272382; doi:10.1371/journal.pmed.1004302)
Supplement: S1 Appendix — Detailed methodological description of the dataset, data sources, and conceptual framework of the study. Additionally, descriptive results by outcomes, results for logistic regression, complementary subgroup results, results for all sensitivity, and triangulation analyses. (DOCX) [file pmed.1004302.s001.docx]

**The impact of primary health care on AIDS: a cohort study of 3.4 million Brazilians**

**Supporting Information**

**Table of contents**

[1. Definitions 2](#_heading=h.30j0zll)

[2. Conceptual framework of the study cohort](#_heading=h.1fob9te) 5

[3. Descriptive analyses by outcomes](#_heading=h.2et92p0) 6

4. Descriptive analyses by outcomes: included and non-included individuals due missing information in independent variables 7

[5. Estimation of logistic regression for the exposure group, by outcome](#_heading=h.tyjcwt) 9

6. Subgroup analysis 10

7. Sensitivity analysis 11

8. Complementary analysis 21

9. Triangulation analysis 28

**1. Definitions**

**The Cadastro Único (CadUnico) database and the 100 Million Brazilians Cohort**

The 100 Million Brazilians Cohort was based on the baseline information of families, during the period from January 1, 2001 to December 31, 2017, who sought to benefit from the Brazilian government's social welfare programs through registration in the Unified Registry for Social Programs (in Portuguese: *Cadastro Único para Programas Sociais* – CadUnico). The CadUnico is an administrative database, to which Brazilians aged 16 or over can apply by registering their personal information (age, sex, skin color, education and others) and household information (household density, familiar income, structural characteristics of the residence and others), as long as they are within one of these categories: (i) belong to a family with a monthly per capita income of up to half a minimum wage; (ii) belong to a family with a total monthly income of up to three minimum wages; (iii) belong to a family with an income greater than three minimum wages, provided that the registration is linked to inclusion in social programs in the three spheres of government; (iv) be the only resident of the household, or; (v) living on the streets (alone or with the family).

Upon registration, individuals receive a unique identifier code and are asked about their socioeconomic characteristics. By the end of 2017, CadÚnico had approximately 114 million individuals registered, which represents around 50% of the Brazilian population. It is a tool that identifies and characterizes especially low-income families, allowing the government to know the socioeconomic status of the poorest half of the population and use this information to determine eligibility for a range of different social welfare programs.^1,2^

In addition to the information from CadÚnico, the Cohort is also composed of health-related databases. For our study, the information used to identify morbidity and mortality from AIDS comes from the National System of Disease Notification (in Portuguese: *Sistema de Informação de Agravos de Notificação - SINAN*) and the Mortality Information System (in Portuguese: *Sistema de Informações Sobre Mortalidade - SIM*).^3^ As standard practice the Ministry of Health updates the HIV/AIDS cases in SINAN through a linkage with other databases like SISCEL (Laboratory Tests Control Systems) and SICLOM (Medication Logistics Control System). SICLOM database covers all people living with HIV/AIDS and receiving ART (antiretroviral therapy), both in public and private health care sectors. SISCEL database, on the other hand, covers only those people living with HIV who had CD4 and viral load tests conducted in public laboratories.^4^

Created by the Center for Integration of Data and Knowledge for Health (in Portuguese: *Centro de Integração de Dados e Conhecimentos para Saúde* - CIDACS/FIOCRUZ)^2^, the Cohort aims to facilitate research and continuous assessment of social determinants and the impact s of social policies and programs in health contexts in Brazil. It has 246 variables with demographic and socioeconomic information at the individual and family levels. The codes and linking algorithms between the databases were built to make efficient and specific links through five identifiers: the date of birth, the municipality of residence, the sex, the name and the mother’s name of each individual presented in each of the databases^2,5,6^. The linkage was performed at the individual level in two stages using the CIDACS-Record Linkage tool ([https://gitHub.com/gcgbarbosa/cidacs-rl](https://github.com/gcgbarbosa/cidacs-rl)). First, the inputs were linked deterministically. In the second step, for cases that were not deterministically linked, they were linked based on a similarity score for all pairwise comparisons, ranging from 0 to 1 - where 1 means that is perfectly similar. The entries with the highest similarity scores (above 0.95) were considered as linked pairs.^7^ The quality of each link for all causes between CadUnico, SINAN e SIM has been extensively evaluated and validated.^8,9^

The variables at the municipal level of the resident individual's baseline were linked to the CadÚnico records by the municipality code and the year of information. The average variables for the period that capture the level of health surveillance and the risk of AIDS endemicity were linked only by the municipal identifier.

**Family Health Strategy (FHS)**

The Family Health Strategy (FHS) was launched in 1994 and has experienced an intense expansion across Brazil’s municipalities. FHS is currently one of the world’s largest communities based primary health care (PHC) programs, involving interdisciplinary health care teams that include a physician, a nurse, a nurse assistant, and four to six full-time community health workers. Family health teams are organized geographically, covering populations of up to 1,000 households, and each FHS team member has defined roles and responsibilities. Moreover, national guidelines help to structure and standardize FHS responses to most health problems. Each community health worker is assigned to approximately 150 households in a geographically delineated micro-area within the catchment area — usually the same micro-area where the community health worker lives. They visit each household within their micro-area at least once per month and collect individual- and household-level data. During each visit they develop health promotion activities, also helping scheduling appointments, check whether prescriptions have been filled and whether patients have been taking their medications regularly. They also ask about changes to household composition, identifying signs of violence and neglect, among other problems. They also actively look for risk factors such as smoking and symptoms of common chronic disease such as hypertension and diabetes.^9^

FHS teams are located near people’s homes to facilitate access and first-contact care. Moreover, the FHS uses geographic paneling through lists of all residents in its catchment area to permit longitudinal care since each team is responsible for a defined population. The care provided by the FHS model is proactive, since community health workers make monthly (or semimonthly) home visits to each enrolled individual in order to seek out problems before patients arrive at the health post.

The annual coverage of the FHS is built from information collected from the Ministry of Health, through the Primary Health Care Information System (Sistema de Informação da Atenção Básica - SIAB), the most updated and consolidated data on the coverage of the FHS in all 5,570 Brazilian municipalities in the period of 2000 to 2018. The FHS exposure variable was calculated as the total number of teams deployed in the municipality that year multiplied by 3,450, which represents the number of people served by each FHS team, divided by the population of the municipality. The FHS coverage is considerably high and showed an increase during the period of the study (from 2007-2015) (Table S1). CadUnico's registrations were linked to the coverage of the municipal FHS by the municipality code.

Regarding the expansion of the FHS, municipal leadership decides to expand FHS teams into areas previously uncovered by existing services or to convert existing public health posts into FHS teams. Once the FHS team is established, they actively seek out and register all residents within their catchment area. There is no evidence that coverage decisions are driven by the burden of any disease (including HIV/AIDS) in the municipality, but instead by exogenous factors such as the mayor’s political party, the municipality’s size and population density, and other structural factors.^10^

**Table S1. Family Health Strategy (FHS) coverage, Brazil, 2007-15**

| Year | 2007 | 2008 | 2009 | 2010 | 2011 | 2012 | 2013 | 2014 | 2015 |
| --- | --- | --- | --- | --- | --- | --- | --- | --- | --- |
| FHS coverage (%) | 48.0 | 51.0 | 51.4 | 53.0 | 54.6 | 55.5 | 57.5 | 61.1 | 62.5 |

Source: <https://egestorab.saude.gov.br/>

**REFERENCES**

1. Sanni Ali M, Ichihara MY, Lopes LC, et al. Administrative data linkage in Brazil: Potentials for health technology assessment. Front Pharmacol 2019;10(SEP):1–20.

2. Barreto ML, Ichihara MY, Pescarini JM, et al. Cohort profile: The 100 Million Brazilian Cohort. 2021;1–12.

3. DATASUS. Ministério da Saúde [Internet]. [cited 2021 Dec 20]; Available from: <https://datasus.saude.gov.br/>

4. Ali et al. Administrative Data Linkage in Brazil: Potentials for Health Technology Assessment. Front. Pharmacol., 23 September 2019. Sec. Drugs Outcomes Research and Policies. Volume 10 - 2019 | [https://doi.org/10.3389/fphar.2019.00984)](https://doi.org/10.3389/fphar.2019.00984)

5. Pita R, Pinto C, Sena S, et al. On the Accuracy and Scalability of Probabilistic Data Linkage over the Brazilian 114 Million Cohort. IEEE J Biomed Heal Informatics 2018;22(2):346–53.

6. Pinto C, Pita R, Barbosa G, et al. Probabilistic Integration of Large Brazilian Socioeconomic and Clinical Databases. Proc - IEEE Symp Comput Med Syst 2017;2017–June:515–20.

7. Almeida, D., Gorender, D., Ichihara, M.Y. et al. Examining the quality of record linkage process using nationwide Brazilian administrative databases to build a large birth cohort. BMC Med Inform Decis Mak 20, 173 (2020). https://doi.org/10.1186/s12911-020-01192-0)

8. Barreto, Marcos1, Alves, André, Sena, Samila, Fiaccone, Rosemeire, Amorim, Leila, Ichihara, Maria Yuri, and Barreto M. Assessing the accuracy of probabilistic record linkage of social and health databases in the 100 million Brazilian cohort. Int J Popul Data Sci 2017;0(August 2016):23889.

9. Pita R, Pinto C, Barreto M, et al. Design and evaluation of probabilistic record linkage methods supporting the Brazilian 100-million cohort initiative. Int J Popul Data Sci 2017;1(1):23889.

10. Andrade MV, Coelho AQ, Xavier Neto M, Carvalho LR de, Atun R, Castro MC. Brazil’s Family Health Strategy: factors associated with programme uptake and coverage expansion over 15 years (1998–2012). Health Policy Plan 2018; 33: 368–80.

**2. Conceptual framework of the study cohort**


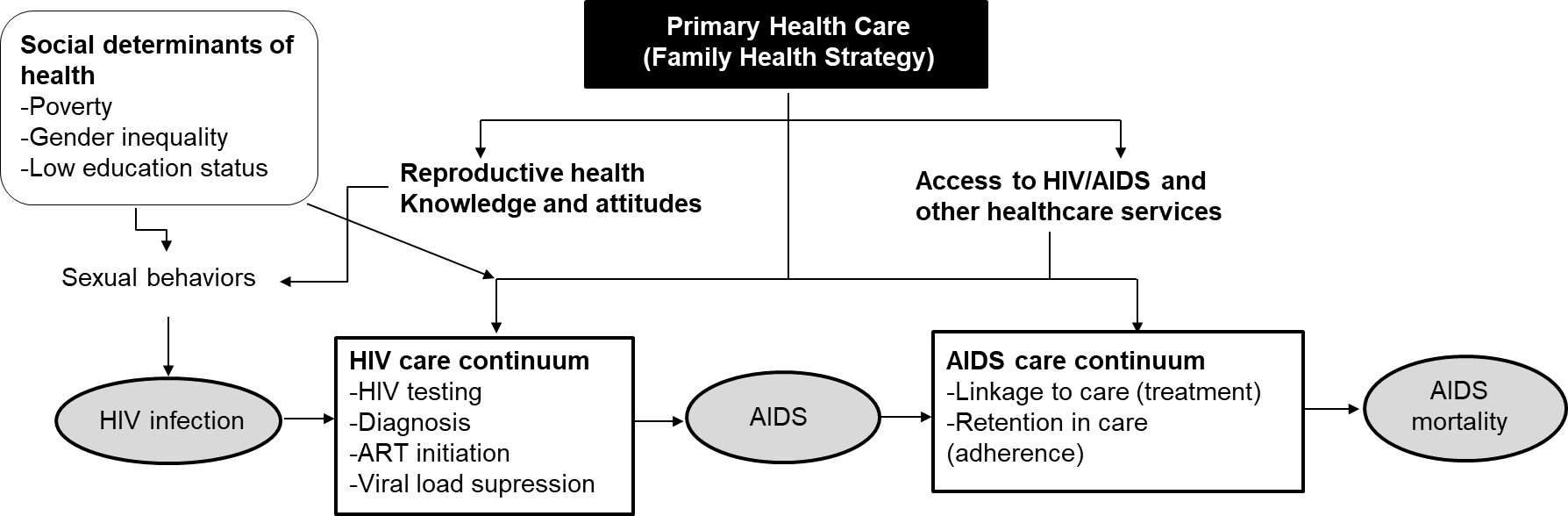


**Figure S1. Conceptual framework of selected structural determinants of HIV infection, AIDS incidence and mortality, and of the hypothesized impacts** **of Primary Health Care and Family Health Strategy on this process.**

**3. Descriptive analyses by outcomes**

**Table S2. Descriptive analysis of AIDS diagnoses and AIDS-related deaths by the Family Health Strategy (FHS) coverage in the study cohort, Brazil, 2007–15.**

|  | **AIDS diagnoses**  **n=2,721** | | **AIDS-related deaths**  **n=819** | |
| --- | --- | --- | --- | --- |
|  | **Unexposed**  **n=673** | **Exposed**  **n=2,048** | **Unexposed**  **n=218** | **Exposed**  **n=601** |
| Sex |  |  |  |  |
| Female | 324 (48.1) | 841 (41.1) | 96 (44.0) | 239 (39.8) |
| Male | 349 (51.9) | 1,207 (58.9) | 122 (56.0) | 362 (60.2) |
| Age (years) |  |  |  |  |
| 13-24 | 113 (16.8) | 537 (26.2) | 21 (9.6) | 95 (15.8) |
| 25-59 | 534 (79.3) | 1,453 (70.9) | 184 (84.4) | 479 (79.7) |
| ≥60 | 26 (3.9) | 58 (2.8) | 13 (6.0) | 27 (4.5) |
| Skin color |  |  |  |  |
| White | 358 (53.2) | 457 (22.3) | 114 (52.3) | 126 (21.0) |
| Asian | 1 (0.15) | 8 (0.4) | 0 (0) | 3 (0.5) |
| Pardo | 233 (34.6) | 1383 (67.5) | 76 (34.9) | 411 (68.4) |
| Black | 80 (11.9) | 193 (9.4) | 28 (12.8) | 60 (10.0) |
| Indigenous | 1 (0.15) | 7 (0.3) | 0 (0) | 1 (0.2) |
| Per capita household expenditures - % MW |  |  |  |  |
| >1 | 44 (6.5) | 63 (3.1) | 9 (4.1) | 16 (2.7) |
| 0.5-1 | 119 (17.7) | 187 (9.1) | 37 (17.0) | 57 (9.5) |
| 0.25-0.49 | 80 (11.9) | 176 (8.6) | 26 (11.9) | 41 (6.8) |
| 0-0.24 | 172 (25.6) | 538 (26.3) | 61 (28.0) | 170 (28.3) |
| Nothing declared | 258 (38.3) | 1,084 (52.9) | 85 (39.0) | 317 (52.7) |
| Education (years of study) |  |  |  |  |
| >9 | 127 (18.9) | 281 (13.7) | 28 (12.8) | 46 (7.6) |
| 4-9 | 265 (39.4) | 653 (31.9) | 86 (39.4) | 183 (30.4) |
| 1-4 | 235 (34.9) | 793 (38.7) | 87 (39.9) | 255 (42.4) |
| No education | 46 (6.8) | 321 (15.7) | 17 (7.8) | 117 (19.5) |
| Water supply |  |  |  |  |
| Public network | 603 (89.6) | 1,389 (67.8) | 200 (91.7) | 407 (67.7) |
| Well, spring or cistern | 70 (10.4) | 659 (32.2) | 18 (8.3) | 194 (32.3) |
| Home construction material |  |  |  |  |
| Brick | 570 (84.7) | 1,436 (70.1) | 187 (85.8) | 432 (71.9) |
| Wook or taipa^1^ | 103 (15.3) | 612 (29.9) | 31 (14.2) | 169 (28.1) |
| Lighting |  |  |  |  |
| Electricity | 576 (85.6) | 1,785 (87.2) | 192 (88.1) | 521 (86.7) |
| No electricity | 97 (14.4) | 263 (12.8) | 26 (11.9) | 80 (13.3) |
| Number of individuals per room |  |  |  |  |
| 1 | 630 (93.6) | 1,958 (95.6) | 203 (93.1) | 576 (95.8) |
| 1-2 | 38 (5.7) | 79 (3.9) | 14 (6.4) | 22 (3.7) |
| >2 | 5 (0.7) | 11 (0.5) | 1 (0.5) | 3 (0.5) |
| Garbage disposal |  |  |  |  |
| Public network | 635 (94.3) | 1,440 (70.3) | 204 (93.6) | 424 (70.6) |
| Burned, buried or another | 38 (5.7) | 608 (29.7) | 14 (6.4) | 177 (29.4) |
| Sewage |  |  |  |  |
| Public network | 549 (81.6) | 551 (26.9) | 182 (83.5) | 163 (27.1) |
| Septic tank | 33 (4.9) | 439 (21.4) | 9 (4.1) | 126 (21.0) |
| Rudimentary cesspit/ditch or another | 91 (13.5) | 1,058 (51.7) | 27 (12.4) | 312 (51.9) |
| Year of entry into the cohort |  |  |  |  |
| 2007 | 226 (33.6) | 878 (42.9) | 81 (37.2) | 261 (43.3) |
| 2008 | 94 (14.0) | 329 (16.1) | 38 (13.8) | 99 (16.5) |
| 2009 | 79 (11.7) | 231 (11.3) | 22 (10.1) | 78 (13.0) |
| 2010 | 91 (13.5) | 174 (8.5) | 30 (13.8) | 44 (7.3) |
| 2011 | 66 (9.8) | 119 (5.8) | 20 (9.2) | 36 (6.0) |
| 2012 | 58 (8.6) | 169 (8.2) | 19 (8.7) | 51 (8.5) |
| 2013 | 26 (3.9) | 82 (4.0) | 10 (4.6) | 17 (2.8) |
| 2014 | 27 (4.0) | 56 (2.7) | 6 (2.7) | 14 (2.3) |
| 2015 | 6 (0.9) | 10 (0.5) | 0 (0) | 1 (0.2) |
|  | Mean (SD) | Mean (SD) | Mean (SD) | Mean (SD) |
| AIDS municipal detection among individuals in the cohort^2^ | 206.0 (167.3) | 154.7 (115.1) | 169.6 (135.4) | 160.2 (129.8) |
| Time receiving Bolsa Família, months | 43.69 (42.8) | 61.87 (51.4) | 40.2 (39.8) | 56.6 (50.6) |
| Municipal unemployment rate, % | 8.22 (2.8) | 8.85 (4.2) | 8.42 (2.8) | 8.95 (4.4) |
| Hospital beds per 1,000 inhabitants | 1.91 (1.3) | 2.08 (2.1) | 2.26 (1.6) | 2.03 (1.9) |

%MW - Proportional to the baseline minimum wage (MW).

1- Taipa is a construction method that consists of using clay and wood to build houses

2- Annual cumulative AIDS incidence for each individual’s municipality of residence from the study cohort

**4. Descriptive analyses by outcomes: included and non-included individuals due missing information in independent variables**

Assumptions about the differences in outcomes between the included and non-included

We can observe that, in both included and non-included individuals for both outcomes, there is a higher proportion of men, people between 25-59 years old and white people.

However, the higher proportion of men and people without declaration of per capita household expenditures among individuals not included highlights the greater social vulnerability in this group. In this way, we can assume that, if individuals not included were included, we would have even clearer and more precise results on social vulnerability.

**Table S3. Descriptive analysis of AIDS diagnoses and AIDS-related deaths by the Family Health Strategy (FHS) coverage in the study cohort: included and non-included individuals due missing information in independent variables, Brazil, 2007–15**.

|  | Individuals included in main analysis | | | | Individuals non-included in main analysis due missing information | | | |
| --- | --- | --- | --- | --- | --- | --- | --- | --- |
|  | AIDS diagnoses  n=2,721 | | AIDS-related deaths  n=819 | | AIDS diagnoses  n=559 | | AIDS-related deaths  n=141 | |
|  | Unexposed  n=673 | Exposed  n=2,048 | Unexposed  n=218 | Exposed  n=601 | Unexposed  n=127 | Exposed  n=432 | Unexposed  n=37 | Exposed  n=104 |
|  | N (%) | N (%) | N (%) | N (%) | N (%) | N (%) | N (%) | N (%) |
| Sex |  |  |  |  |  |  |  |  |
| Female | 324 (48.1) | 841 (41.1) | 96 (44.0) | 239 (39.8) | 46 (36.2) | 142 (32.8) | 11 (29.7) | 33 (31.7) |
| Male | 349 (51.9) | 1,207 (58.9) | 122 (56.0) | 362 (60.2) | 81 (63.8) | 290 (67.1) | 26 (70.3) | 71 (68.3) |
| Missing | - | - | - | - | 0 (0) | 0 (0) | 0 (0) | 0 (0) |
| Age (years) |  |  |  |  |  |  |  |  |
| 13-24 | 113 (16.8) | 537 (26.2) | 21 (9.6) | 95 (15.8) | 26 (20.5) | 127 (29.4) | 4 (10.8) | 19 (18.3) |
| 25-59 | 534 (79.3) | 1,453 (70.9) | 184 (84.4) | 479 (79.7) | 95 (74.8) | 297 (68.8) | 29 (78.4) | 85 (81.7) |
| ≥60 | 26 (3.9) | 58 (2.8) | 13 (6.0) | 27 (4.5) | 6 (4.7) | 8 (1.8) | 4 (10.8) | 0 (0) |
| Missing | - | - | - | - | 0 (0) | 0 (0) | 0 (0) | 0 (0) |
| Skin color |  |  |  |  |  |  |  |  |
| White | 358 (53.2) | 457 (22.3) | 114 (52.3) | 126 (21.0) | 43 (33.9) | 86 (19.9) | 12 (32.4) | 23 (22.1) |
| Asian | 1 (0.15) | 8 (0.4) | 0 (0) | 3 (0.5) | 0 (0) | 1 (0.2) | 0 (0) | 0 (0) |
| Pardo | 233 (34.6) | 1383 (67.5) | 76 (34.9) | 411 (68.4) | 27 (21.3) | 219 (50.7) | 8 (21.6) | 55 (52.9) |
| Black | 80 (11.9) | 193 (9.4) | 28 (12.8) | 60 (10.0) | 8 (6.3) | 19 (4.4) | 4 (10.8) | 4 (3.8) |
| Indigenous | 1 (0.15) | 7 (0.3) | 0 (0) | 1 (0.2) | 1 (0.8) | 0 (0) | 0 (0) | 0 (0) |
| Missing | - | - | - | - | 48 (37.8) | 107 (24.8) | 13 (35.1) | 22 (21.1) |
| Per capita household expenditures - % MW |  |  |  |  |  |  |  |  |
| >1 | 44 (6.5) | 63 (3.1) | 9 (4.1) | 16 (2.7) | 7 (5.5) | 10 (2.3) | 3 (8.1) | 2 (1.9) |
| 0.5-1 | 119 (17.7) | 187 (9.1) | 37 (17.0) | 57 (9.5) | 10 (7.9) | 36 (8.3) | 4 (10.8) | 10 (9.6) |
| 0.25-0.49 | 80 (11.9) | 176 (8.6) | 26 (11.9) | 41 (6.8) | 14 (11.0) | 36 (8.3) | 5 (13.5) | 11 (10.6) |
| 0-0.24 | 172 (25.6) | 538 (26.3) | 61 (28.0) | 170 (28.3) | 33 (26.0) | 106 (24.5) | 6 (16.2) | 31 (29.8) |
| Nothing declared | 258 (38.3) | 1,084 (52.9) | 85 (39.0) | 317 (52.7) | 63 (49.6) | 244 (56.5) | 19 (51.3) | 50 (48.1) |
| Missing | - | - | - | - | 0 (0) | 0 (0) | 0 (0) | 0 (0) |
| Education (years of study) |  |  |  |  |  |  |  |  |
| >9 | 127 (18.9) | 281 (13.7) | 28 (12.8) | 46 (7.6) | 12 (9.5) | 33 (7.6) | 1 (2.7) | 4 (3.8) |
| 4-9 | 265 (39.4) | 653 (31.9) | 86 (39.4) | 183 (30.4) | 9 (7.1) | 32 (7.4) | 4 (10.8) | 7 (6.7) |
| 1-4 | 235 (34.9) | 793 (38.7) | 87 (39.9) | 255 (42.4) | 17 (13.4) | 56 (13.0) | 7 (18.9) | 15 (14.4) |
| No education | 46 (6.8) | 321 (15.7) | 17 (7.8) | 117 (19.5) | 1 (0.8) | 28 (6.5) | 1 (2.7) | 11 (10.6) |
| Missing | - | - | - | - | 88 (69.3) | 283 (65.5) | 24 (64.9) | 67 (64.4) |
| Water supply |  |  |  |  |  |  |  |  |
| Public network | 603 (89.6) | 1,389 (67.8) | 200 (91.7) | 407 (67.7) | 102 (80.3) | 262 (60.6) | 25 (67.6) | 59 (56.7) |
| Well, spring or cistern | 70 (10.4) | 659 (32.2) | 18 (8.3) | 194 (32.3) | 11 (8.7) | 115 (26.6) | 4 (10.8) | 27 (26.0) |
| Missing | - | - | - | - | 14 (11.0) | 55 (12.7) | 8 (21.6) | 18 (17.3) |
| Home construction material |  |  |  |  |  |  |  |  |
| Brick | 570 (84.7) | 1,436 (70.1) | 187 (85.8) | 432 (71.9) | 102 (80.3) | 264 (61.1) | 27 (73.0) | 63 (60.6) |
| Wook or taipa1 | 103 (15.3) | 612 (29.9) | 31 (14.2) | 169 (28.1) | 11 (8.7) | 113 (26.2) | 2 (5.4) | 23 (22.1) |
| Missing | - | - | - | - | 14 (11.0) | 55 (12.7) | 8 (21.6) | 18 (17.3) |
| Lighting |  |  |  |  |  |  |  |  |
| Electricity | 576 (85.6) | 1,785 (87.2) | 192 (88.1) | 521 (86.7) | 100 (78.7) | 333 (77.1) | 23 (62.2) | 74 (71.1) |
| No electricity | 97 (14.4) | 263 (12.8) | 26 (11.9) | 80 (13.3) | 13 (10.2) | 44 (10.2) | 6 (16.2) | 12 (11.5) |
| Missing | - | - | - | - | 14 (11.0) | 55 (12.7) | 8 (21.6) | 18 (17.3) |
| Number of individuals per room |  |  |  |  |  |  |  |  |
| 1 | 630 (93.6) | 1,958 (95.6) | 203 (93.1) | 576 (95.8) | 105 (82.7) | 364 (84.3) | 26 (70.3) | 84 (80.8) |
| 1-2 | 38 (5.7) | 79 (3.9) | 14 (6.4) | 22 (3.7) | 5 (3.9) | 10 (2.3) | 1 (2.7) | 1 (1.0) |
| >2 | 5 (0.7) | 11 (0.5) | 1 (0.5) | 3 (0.5) | 3 (2.4) | 3 (0.7) | 2 (5.4) | 1 (1.0) |
| Missing | - | - | - | - | 14 (11.0) | 55 (12.7) | 8 (21.6) | 18 (17.3) |
| Garbage disposal |  |  |  |  |  |  |  |  |
| Public network | 635 (94.3) | 1,440 (70.3) | 204 (93.6) | 424 (70.6) | 110 (86.6) | 252 (58.3) | 28 (75.7) | 57 (54.8) |
| Burned, buried or another | 38 (5.7) | 608 (29.7) | 14 (6.4) | 177 (29.4) | 3 (2.4) | 125 (28.9) | 1 (2.7) | 29 (27.9) |
| Missing | - | - | - | - | 14 (11.0) | 55 (12.7) | 8 (21.6) | 18 (17.3) |
| Sewage |  |  |  |  |  |  |  |  |
| Public network | 549 (81.6) | 551 (26.9) | 182 (83.5) | 163 (27.1) | 95 (74.8) | 93 (21.5) | 24 (64.9) | 23 (22.1) |
| Septic tank | 33 (4.9) | 439 (21.4) | 9 (4.1) | 126 (21.0) | 4 (3.1) | 90 (20.8) | 1 (2.7) | 22 (21.1) |
| Rudimentary cesspit/ditch or another | 91 (13.5) | 1,058 (51.7) | 27 (12.4) | 312 (51.9) | 13 (10.2) | 140 (32.4) | 4 (10.8) | 30 (28.8) |
| Missing | - | - | - | - | 15 (11.1) | 109 (25.2) | 8 (21.6) | 29 (27.9) |
| Year of entry into the cohort |  |  |  |  |  |  |  |  |
| 2007 | 226 (33.6) | 878 (42.9) | 81 (37.2) | 261 (43.3) | 39 (30.7) | 168 (38.0) | 15 (40.5) | 33 (31.7) |
| 2008 | 94 (14.0) | 329 (16.1) | 38 (13.8) | 99 (16.5) | 22 (17.3) | 80 (18.5) | 6 (16.2) | 19 (18.3) |
| 2009 | 79 (11.7) | 231 (11.3) | 22 (10.1) | 78 (13.0) | 24 (18.9) | 42 (9.7) | 3 (8.1) | 15 (14.4) |
| 2010 | 91 (13.5) | 174 (8.5) | 30 (13.8) | 44 (7.3) | 10 (7.9) | 23 (5.3) | 0 (0) | 4 (3.8) |
| 2011 | 66 (9.8) | 119 (5.8) | 20 (9.2) | 36 (6.0) | 4 (3.1) | 20 (4.6) | 3 (8.1) | 3 (2.9) |
| 2012 | 58 (8.6) | 169 (8.2) | 19 (8.7) | 51 (8.5) | 5 (3.9) | 34 (7.9) | 3 (8.1) | 10 (9.6) |
| 2013 | 26 (3.9) | 82 (4.0) | 10 (4.6) | 17 (2.8) | 19 (15.0) | 51 (11.8) | 6 (16.2) | 15 (14.4) |
| 2014 | 27 (4.0) | 56 (2.7) | 6 (2.7) | 14 (2.3) | 3 (2.4) | 13 (3.0) | 0 (0) | 3 (2.9) |
| 2015 | 6 (0.9) | 10 (0.5) | 0 (0) | 1 (0.2) | 1 (0.8) | 5 (1.2) | 1 (2.7) | 2 (1.9) |
| Missing | - | - | - | - | 0 (0) | 0 (0) | 0 (0) | 0 (0) |
|  | Mean (SD) | Mean (SD) | Mean (SD) | Mean (SD) | Mean (SD) | Mean (SD) | Mean (SD) | Mean (SD) |
| AIDS municipal detection among individuals in the cohort2 | 206.0 (167.3) | 154.7 (115.1) | 169.6 (135.4) | 160.2 (129.8) | 172.0 (145.2) | 140.4 (95.2) | 111.4 (59.5) | 144.7 (95.7) |
| Time receiving Bolsa Família, months | 43.7 (42.8) | 61.9 (51.4) | 40.2 (39.8) | 56.6 (50.6) | 44.6 (44.9) | 57.2 (53.2) | 48.9 (48.5) | 52.5 (54.5) |
| Municipal unemployment rate, % | 8.2 (2.8) | 8.8 (4.2) | 8.42 (2.8) | 8.9 (4.4) | 8.2 (3.0) | 8.5 (4.4) | 8.2 (3.2) | 9.5 (4.9) |
| Hospital beds per 1,000 inhabitants | 1.9 (1.3) | 2.1 (2.1) | 2.3 (1.6) | 2.0 (1.9) | 2.1 (1.4) | 2.2 (2.2) | 2.5 (1.9) | 2.1 (2.8) |

%MW - Proportional to the baseline minimum wage (MW).

1- Taipa is a construction method that consists of using clay and wood to build houses

2- Annual cumulative AIDS incidence for each individual’s municipality of residence from the study cohort

**5. Estimation of logistic regression for the exposure group, by outcome**

**Table S4. Prediction models using logistic regression for residence in municipalities with Family Health Strategy coverage (<20% and 100%) in the 100 Million Brazilians Cohort, by AIDS outcomes, Brazil, 2007-15**

|  | **AIDS Incidence**  **n=3,588,770** | **AIDS Mortality**  **n=3,588,770** |
| --- | --- | --- |
|  | **OR (95% CI)** | **OR (95% CI)** |
| Sex |  |  |
| Female | 1 | 1 |
| Male | 1.24 (1.23-1.25) | 1.24 (1.23-1.25) |
| Age (years) |  |  |
| 13-24 | 1 | 1 |
| 25-59 | 0.86 (0.85-0.87) | 0.86 (0.85-0.87) |
| >=60 | 1.10 (1.09-1.12) | 1.10 (1.09-1.12) |
| Skin color |  |  |
| White | 1 | 1 |
| Asian | 2.29 (2.18-2.40) | 2.29 (2.18-2.40) |
| Pardo | 2.51 (2.49-2.52) | 2.51 (2.49-2.52) |
| Black | 1.36 (1.35-1.38) | 1.36 (1.35-1.38) |
| Indigenous | 1.36 (1.35-1.38) | 1.36 (1.35-1.38) |
| Education (years of study) |  |  |
| >9 | 1 | 1 |
| 4-9 | 0.86 (0.85-0.86) | 0.86 (0.85-0.86) |
| 1-4 | 1.15 (1.14-1.16) | 1.15 (1.14-1.16) |
| No education | 2.05 (2.03-2.07) | 2.05 (2.03-2.08) |
| Per capita household expenditures - % MW |  |  |
| >1 | 1 | 1 |
| 0.5-1 | 1.59 (1.57-1.60) | 1.59 (1.57-1.60) |
| 0.25-0.49 | 2.41 (2.38-2.44) | 2.41 (2.38-2.44) |
| 0-0.24 | 2.51 (2.48-2.54) | 2.51 (2.48-2.54) |
| Nothing declared | 3.54 (3.50-3.60) | 3.54 (3.50-3.59) |
| Home construction material |  |  |
| Brick | 1 | 1 |
| Wood or taipa^1^ | 3.69 (3.65-3.73) | 3.65 (3.65-3.73) |
| Number of individuals per room |  |  |
| 1 | 1 | 1 |
| 1-2 | 0.31 (0.30-0.31) | 0.31 (0.30-0.31) |
| >2 | 0.23 (0.22-0.24) | 0.23 (0.22-0.24) |
| Year of entry into the cohort |  |  |
| 2007 | 1 | 1 |
| 2008 | 0.78 (0.77-0.79) | 0.78 (0.77-0.79) |
| 2009 | 0.79 (0.78-0.80) | 0.79 (0.78-0.80) |
| 2010 | 0.62 (0.61-0.63) | 0.62 (0.61-0.63) |
| 2011 | 0.83 (0.82-0.84) | 0.83 (0.82-0.84) |
| 2012 | 1.19 (1.17-1.20) | 1.19 (1.17-1.20) |
| 2013 | 1.37 (1.35-1.39) | 1.37 (1.35-1.39) |
| 2014 | 1.10 (1.09-1.12) | 1.10 (1.09-1.12) |
| 2015 | 0.87 (0.86-0.88) | 0.87 (0.86-0.88) |
| AIDS municipal incidence among individuals in the cohort^2^ | 0.99 (0.99-0.99) | 0.99 (0.99-0.99) |
| Time receiving Bolsa Família, months | 1.00 (1.00-1.00) | 1.00 (1.00-1.00) |

OR: Odds Ratio; CI: Confidence interval

1- Taipa is a construction method that consists of using clay and wood to build houses

2- Annual cumulative AIDS detection for each individual’s municipality of residence from the study cohort

**6. Complementary subgroup analysis**

**AIDS incidence and AIDS mortality by adequate municipal quality of vital information**

**Table S5. Inverse probability of treatment weighting (IPTW) Poisson regression models, adjusted for all demographic and socioeconomic variables, for the association between AIDS incidence and mortality and Family Health Strategy (FHS) coverage by municipal quality of vital information, Brazil, 2007–15**

| **Municipal quality of vital information** | **AIDS Incidence** | **AIDS Mortality** |
| --- | --- | --- |
|  | **RRa (95%CI)** | **RRa (95%CI)** |
| Adequate | 0.78 (0.69-0.87) | 0.52 (0.43-0.63) |

RRa=rate risk adjusted for sex, age, skin color, education, per capita household expenditures, home construction material, number of people per room, year of entry into the cohort, time receiving Bolsa Família (in months), AIDS municipal rate in the cohort, water supply, lighting, sewage, garbage disposal, municipal unemployment rate, and hospital beds per 1,000 inhabitants.

CI=Confidence Interval

**7. Sensitivity analysis**

**Sensitivity analysis using other Family Health Strategy (FHS) coverage levels**

**Table S6. Absolute number of municipalities, AIDS diagnoses and AIDS-related deaths by different coverage levels of the Family Health Strategy (FHS), Brazil, 2007–15**.

|  | **FHS coverage**  **0%** | **FHS coverage**  **<10%** | **FHS coverage**  **<20%** | **FHS coverage**  **100%** |
| --- | --- | --- | --- | --- |
|  | n | n | n | n |
| AIDS diagnoses | 97 | 159 | 673 | 2,048 |
| AIDS-related deaths | 30 | 50 | 218 | 601 |
| Municipalities | 63 | 71 | 104 | 1,960 |

**Table S7. Sensitivity analysis for AIDS incidence and mortality rates using other coverage levels of the Family Health Strategy (FHS), Brazil, 2007–2015**

| **Outcome** | **Poisson IPTW regression analysis** | | |  |
| --- | --- | --- | --- | --- |
|  | **FHS coverage**  **<20%** | **FHS coverage**  **0%** | **FHS coverage**  **<10%** | **FHS coverage**  **20-70%** |
|  | **RRa (CI 95%)** | **RRa (CI 95%)** | **RRa (CI 95%)** | **RRa (CI 95%)** |
| AIDS Incidence | 0.76 (0.68-0.84) | 0.74 (0.54-1.02) | 0.68 (0.52-0.89) | 0.98 (0.91-1.06) |
| AIDS Mortality | 0.68 (0.56-0.82) | 0.53 (0.26-1.08) | 0.46 (0.24-0.88) | 0.91 (0.79-1.05) |

RRa=rate risk adjusted by sex, age, skin color, education, per capita household expenditures, home construction material, number of people per room, year of entry into the cohort, time receiving Bolsa Família (in months), AIDS municipal incidence in the cohort, water supply, lighting, sewage, garbage disposal, municipal unemployment rate, hospital beds per 1,000 inhabitants

CI=Confidence Interval

**Table S8. Sensitivity analysis for AIDS incidence and mortality rates using all spectrum of the Family Health Strategy (FHS) coverage, Brazil, 2007–2015**

| Outcome | **Poisson IPTW regression analysis** | | | |
| --- | --- | --- | --- | --- |
|  | **FHS coverage**  **<20%** | **FHS coverage**  **20-70%** | **FHS coverage**  **71%-99%** | **FHS coverage**  **100%** |
|  |  | **RRa (CI 95%)** | **RRa (CI 95%)** | **RRa (CI 95%)** |
| AIDS Incidence | 1 (ref) | 1.06 (0.98-1.14) | 0.83 (0.76-0.92) | 0.69 (0.63-0.75) |
| AIDS Mortality | 1 (ref) | 0.97 (0.84-1.12) | 0.81 (0.68-0.97) | 0.63 (0.53-0.74) |

RRa=rate risk adjusted by sex, age, skin color, education, per capita household expenditures, home construction material, number of people per room, year of entry into the cohort, time receiving Bolsa Família (in months), AIDS municipal incidence in the cohort, water supply, lighting, sewage, garbage disposal, municipal unemployment rate, hospital beds per 1,000 inhabitants

CI=Confidence Interval

**Sensitivity analyses using other using other methods (different by main analysis)**

**Table S9. Sensitivity analysis for AIDS incidence and mortality rates using other methods, Brazil, 2007-15**

| Outcomes | **Poisson IPTW regression analysis** | | | | **Poisson regression**  **(without IPTW)** | **Negative binomial regression** |
| --- | --- | --- | --- | --- | --- | --- |
|  | Main **analysis used in the study** | **Without municipal**  **AIDS endemicity** | **Without municipal covariates** | **Account for clustering within the municipality** |  |  |
|  | **RRa^1^**  **(CI 95%)** | **RRa^2^**  **(CI 95%)** | **RRa^3^**  **(CI 95%)** | **RRa^1^**  **(CI 95%)** | **RRa^1^**  **(CI 95%)** | **IRRa^1^**  **(CI 95%)** |
| AIDS Incidence | 0.76  (0.68-0.84) | 0.68  (0.61-0.75) | 0.93  (0.83-1.06) | 0.76  (0.63-0.90) | 0.83  (0.75-0.92) | 0.84  (0.74-0.95) |
| AIDS Mortality | 0.68  (0.56-0.82) | 0.60  (0.50-0.72) | 0.75  0.60-0.94) | 0.68  (0.49-0.94) | 0.73  (0.61-0.88) | 0.69  (0.55-0.86) |

1-RRa and IRRa=rate risk or incidence rate ratio adjusted by sex, age, skin color, education, per capita household expenditures, home construction material, number of people per room, year of entry into the cohort, time receiving Bolsa Família (in months), AIDS municipal rate in the cohort, water supply, lighting, sewage, garbage disposal, municipal unemployment rate, hospital beds per 1,000 inhabitants

2-RRa=rate risk adjusted by sex, age, skin color, education, per capita household expenditures, home construction material, number of people per room, year of entry into the cohort, time receiving Bolsa Família (in months), water supply, lighting, sewage, garbage disposal, municipal unemployment rate, hospital beds per 1,000 inhabitants

3-RRa=rate risk adjusted by sex, age, skin color, education, per capita household expenditures, home construction material, number of people per household, year of entry into the cohort, time receiving Bolsa Família (in months), water supply, lighting, sewage, garbage disposal

CI=Confidence Interval; IPTW=Inverse probability of treatment weighting

**Negative control analysis using external causes related deaths and its association with Family Health Strategy (FHS)**

**Table S10. Descriptive analyses of external causes-related deaths by Family Health Strategy, Brazil, 2007-15**

| **Unexposed** | **Exposed** | **Total** |
| --- | --- | --- |
| 2,567 (13.4) | 16,641 (86.6) | 19,208 (100.0) |

**Table S11. Inverse probability of treatment weighting (IPTW) Poisson regression models, adjusted for all demographic and socioeconomic variables, for the association between external causes related deaths and Family Health Strategy (FHS) coverage in the study cohort, Brazil, 2007–15**

| **Variables** | **External causes related deaths n=3,435,068** |
| --- | --- |
|  | **RRa (CI 95%)** |
| FHS coverage |  |
| <20% | 1 |
| 100% | 1.01 (0.96-1.07) |
| Sex |  |
| Female | 1 |
| Male | 5.32 (5.08-5.57) |
| Age (years) |  |
| 13-24 | 1 |
| 25-59 | 0.90 (0.75-1.32) |
| ≥60 | 0.82 (0.77-0.88) |
| Skin color |  |
| White | 1 |
| Asian | 0.99 (0.75-1.32) |
| Pardo | 1.10 (1.06-1.15) |
| Black | 1.15 (1.08-1.23) |
| Indigenous | 1.16 (0.91-1.47) |
| Education (years of study) |  |
| >9 | 1 |
| 4-9 | 1.52 (1.43-1.61) |
| 1-4 | 1.69 (1.60-1.79) |
| No education | 1.71 (1.60-1.83) |
| Per capita expenditures - % MW |  |
| >1 | 1 |
| 0.5-1 | 1.00 (0.91-1.10) |
| 0.25-0.49 | 1.11 (1.01-1.22) |
| 0-0.24 | 1.16 (1.06-1.28) |
| Nothing declared | 1.24 (1.12-1.36) |
| Home construction material |  |
| Brick | 1 |
| Wood or taipa^1^ | 0.96 (0.92-1.00) |
| Water supply |  |
| Public network | 1 |
| Well, spring or cistern | 0.98 (0.94-1.02) |
| Lighting |  |
| Electricity | 1 |
| No electricity | 1.05 (1.00-1.11) |
| Garbage disposal |  |
| Public network | 1 |
| Burned, buried or another | 0.82 (0.79-0.86) |
| Sewage |  |
| Public network | 1 |
| Septic tank | 1.05 (1.00-1.10) |
| Rudimentary cesspit/ditch or another | 1.05 (1.00-1.09) |
| Number of individuals per room |  |
| 1 | 1 |
| 1-2 | 1.04 (0.97-1.12) |
| >2 | 1.24 (1.05-1.47) |
| Year of entry into the cohort |  |
| 2007 | 1 |
| 2008 | 1.07 (1.02-1.12) |
| 2009 | 1.08 (1.03-1.14) |
| 2010 | 1.03 (0.96-1.10) |
| 2011 | 1.08 (1.00-1.17) |
| 2012 | 1.06 (0.97-1.14) |
| 2013 | 1.01 (0.92-1.12) |
| 2014 | 1.08 (0.97-1.21) |
| 2015 | 1.17 (0.96-1.42) |
| AIDS municipal incidence among individuals in the cohort^2^ | 1.00 (1.00-1.00) |
| Time receiving Bolsa Família, months | 1.00 (1.00-1.00) |
| Municipal unemployment rate | 1.01 (1.01-1.01) |
| Hospital beds per 1,000 inhabitants | 1.00 (1.00-1.01) |

RRa=rate risk adjusted by sex, age, skin color, education, per capita expenditures, home construction material, number of people per room, year of entry into the cohort, time receiving Bolsa Família (in months), AIDS municipal incidence in the cohort, water supply, lighting, sewage, garbage disposal, municipal unemployment rate, hospital beds per 1,000 inhabitants

1- Taipa is a construction method that consists of using clay and wood to build houses

2- Annual cumulative AIDS incidence for each individual’s municipality of residence from the study cohort

CI=Confidence Interval; FHS= Family Health Strategy; %MW - Proportional to the baseline minimum wage

**IPTW Poisson regression analysis including marital status as covariate**

**Table S12. Inverse probability of treatment weighting Poisson (IPTW) regression models, adjusted for all demographic and socioeconomic variables, for the association between AIDS incidence and Family Health Strategy (FHS) coverage using marital status in the study cohort, Brazil, 2007–15**

| **Variables** | **AIDS incidence**  **n=3,435,068** |
| --- | --- |
|  | **RRa (CI 95%)** |
| FHS coverage |  |
| <20% | 1 |
| 100% | 0.76 (0.69-0.85) |
| Sex |  |
| Female | 1 |
| Male | 1.17 (1.08-1.27) |
| Age (years) |  |
| 13-24 | 1 |
| 25-59 | 2.29 (2.07-2.53) |
| ≥60 | 0.49 (0.38-0.63) |
| Skin color |  |
| White | 1 |
| Asian | 1.42 (0.73-2.74) |
| Pardo | 1.33 (1.21-1.47) |
| Black | 1.65 (1.43-1.92) |
| Indigenous | 1.04 (0.52-2.09) |
| Marital status |  |
| Married | 1 |
| Divorced | 2.57 (2.06-3.21) |
| Single | 2.49 (2.18-2.85) |
| Missing | 2.40 (1.91-3.01) |
| Education (years of study) |  |
| >9 | 1 |
| 4-9 | 1.52 (1.34-1.72) |
| 1-4 | 1.43 (1.26-1.62) |
| No education | 1.26 (1.07-1.47) |
| Per capita expenditures - % MW |  |
| >1 | 1 |
| 0.5-1 | 1.47 (1.17-1.84) |
| 0.25-0.49 | 1.83 (1.44-2.32) |
| 0-0.24 | 2.17 (1.69-2.78) |
| Nothing declared | 2.30 (1.78-2.98) |
| Home construction material |  |
| Brick | 1 |
| Wood or taipa^1^ | 1.14 (1.03-1.26) |
| Water supply |  |
| Public network | 1 |
| Well, spring or cistern | 0.90 (0.80-1.00) |
| Lighting |  |
| Electricity | 1 |
| No electricity | 1.15 (1.01-1.30) |
| Garbage disposal |  |
| Public network | 1 |
| Burned, buried or another | 0.66 (0.58-0.75) |
| Sewage |  |
| Public network | 1 |
| Septic tank | 0.80 (0.71-0.91) |
| Rudimentary cesspit/ditch or another | 0.89 (0.80-0.99) |
| Number of individuals per room |  |
| 1 | 1 |
| 1-2 | 1.00 (0.81-1.24) |
| >2 | 0.78 (0.46-1.30) |
| Year of entry into the cohort |  |
| 2007 | 1 |
| 2008 | 1.09 (0.97-1.23) |
| 2009 | 0.99 (0.86-1.13) |
| 2010 | 1.08 (0.93-1.25) |
| 2011 | 0.94 (0.74-1.20) |
| 2012 | 1.06 (0.83-1.34) |
| 2013 | 1.17 (0.89-1.54) |
| 2014 | 1.20 (0.89-1.62) |
| 2015 | 0.82 (0.48-1.39) |
| AIDS municipal incidence among individuals in the cohort^2^ | 1.00 (1.00-1.00) |
| Time receiving Bolsa Família, months | 1.00 (1.00-1.00) |
| Municipal unemployment rate | 1.00 (0.99-1.01) |
| Hospital beds per 1,000 inhabitants | 1.05 (1.03-1.07) |

RRa=rate risk adjusted by sex, age, skin color, education, per capita expenditures, home construction material, number of people per room, year of entry into the cohort, time receiving Bolsa Família (in months), AIDS municipal incidence in the cohort, water supply, lighting, sewage, garbage disposal, municipal unemployment rate, hospital beds per 1,000 inhabitants

1- Taipa is a construction method that consists of using clay and wood to build houses

2- Annual cumulative AIDS incidence for each individual’s municipality of residence from the study cohort

CI=Confidence Interval; FHS= Family Health Strategy; %MW - Proportional to the baseline minimum wage

**Table S13. Inverse probability of treatment weighting (IPTW) Poisson regression models, adjusted for all demographic and socioeconomic variables, for the association between AIDS mortality and Family Health Strategy (FHS) coverage using marital status in the study cohort, Brazil, 2007–15**

| **Variables** | **AIDS mortality**  **n=3,435,068** |
| --- | --- |
|  | **RRa (CI 95%)** |
| FHS coverage |  |
| <20% | 1 |
| 100% | 0.68 (0.56-0.83) |
| Sex |  |
| Female | 1 |
| Male | 1.25 (1.06-1.46) |
| Age (years) |  |
| 13-24 | 1 |
| 25-59 | 4.18 (3.38-5.17) |
| ≥60 | 1.15 (0.76-1.74) |
| Skin color |  |
| White | 1 |
| Asian | 1.71 (0.55-5.36) |
| Pardo | 1.45 (1.20-1.76) |
| Black | 1.74 (1.35-2.25) |
| Indigenous | 0.48 (0.07-3.47) |
| Marital status |  |
| Married | 1 |
| Divorced | 3.64 (2.58-5.16) |
| Single | 2.88 (2.28-3.64) |
| Missing | 1.92 (1.20-3.06) |
| Education (years of study) |  |
| >9 | 1 |
| 4-9 | 2.38 (1.83-3.10) |
| 1-4 | 2.32 (1.78-3.02) |
| No education | 2.20 (1.60-3.03) |
| Per capita expenditures - % MW |  |
| >1 | 1 |
| 0.5-1 | 1.89 (1.19-2.99) |
| 0.25-0.49 | 2.01 (1.23-3.30) |
| 0-0.24 | 2.63 (1.55-4.47) |
| Nothing declared | 2.63 (1.53-4.53) |
| Home construction material |  |
| Brick | 1 |
| Wood or taipa^1^ | 1.05 (0.87-1.28) |
| Water supply |  |
| Public network | 1 |
| Well, spring or cistern | 0.85 (0.69-1.05) |
| Lighting |  |
| Electricity | 1 |
| No electricity | 1.11 (0.88-1.41) |
| Garbage disposal |  |
| Public network | 1 |
| Burned, buried or another | 0.74 (0.58-0.93) |
| Sewage |  |
| Public network | 1 |
| Septic tank | 0.81 (0.64-1.01) |
| Rudimentary cesspit/ditch or another | 0.88 (0.73-1.07) |
| Number of individuals per room |  |
| 1 | 1 |
| 1-2 | 1.28 (0.84-1.93) |
| >2 | 0.74 (0.27-2.01) |
| Year of entry into the cohort |  |
| 2007 | 1 |
| 2008 | 0.96 (0.77-1.19) |
| 2009 | 0.97 (0.76-1.22) |
| 2010 | 0.90 (0.68-1.18) |
| 2011 | 1.11 (0.70-1.76) |
| 2012 | 1.21 (0.78-1.89) |
| 2013 | 1.17 (0.68-2.02) |
| 2014 | 1.14 (0.62-2.07) |
| 2015 | 0.19 (0.02-1.42) |
| AIDS municipal incidence among individuals in the cohort^2^ | 1.00 (1.00-1.00) |
| Time receiving Bolsa Família, months | 0.99 (0.99-1.00) |
| Municipal unemployment rate | 1.00 (0.98-1.02) |
| Hospital beds per 1,000 inhabitants | 1.06 (1.03-1.09) |

RRa=rate risk adjusted by sex, age, skin color, education, per capita expenditures, home construction material, number of people per room, year of entry into the cohort, time receiving Bolsa Família (in months), AIDS municipal incidence in the cohort, water supply, lighting, sewage, garbage disposal, municipal unemployment rate, hospital beds per 1,000 inhabitants

1- Taipa is a construction method that consists of using clay and wood to build houses

2- Annual cumulative AIDS incidence for each individual’s municipality of residence from the study cohort

CI=Confidence Interval; FHS= Family Health Strategy; %MW - Proportional to the baseline minimum wage

**Type of likely exposure among people living with AIDS and who die by AIDS**

**Table S14. Type of likely exposure among people living with AIDS by Family Health**  **Strategy coverage, Brazil, 2007-15**

| **Likely exposure** | **Unexposed**  **n=673** | **Exposed**  **n=2,048** | **Men**  **n=1,556** | **Women**  **n=1,165** | **Total**  **n=2,721** |
| --- | --- | --- | --- | --- | --- |
| Accident | 0 (0.0) | 1 (0.05) | 0 (0.0) | 1 (0.08) | 1 (0.04) |
| Heterossexual | 360 (53.5) | 1,232 (60.1) | 735 (47.2) | 857 (73.6) | 1,592 (58.5) |
| Homossexual | 57 (8.5) | 156 (7.6) | 203 (13.0) | 10 (0,9) | 213 (7.8) |
| Bissexual | 22 (3.3) | 71 (3.5) | 89 (5.7) | 4 (0.4) | 93 (3.4) |
| Drug use | 24 (3.6) | 37 (1.8) | 46 (3.0) | 15 (1.3) | 61 (2.2) |
| Missing | 210 (31.2) | 551 (26.9) | 483 (31.0) | 278 (23.9) | 761 (28.0) |

**Table S15. Type of likely exposure among people who die by AIDS, Brazil, 2007-15**

| **Likely exposure** | **Unexposed**  **n=218** | **Exposed**  **n=601** | **Men**  **n=484** | **Women**  **n=335** | **Total**  **n=819** |
| --- | --- | --- | --- | --- | --- |
| Heterossexual | 49 (22.5) | 217 (36.1) | 142 (29.3) | 124 (37.1) | 266 (32.5) |
| Homossexual | 3 (1.4) | 14 (2.3) | 14 (2.9) | 3 (0.9) | 17 (2.1) |
| Bissexual | 1 (0.4) | 9 (1.5) | 10 (2.1) | 0 (0.0) | 10 (1.2) |
| Drug use | 8 (3.7) | 7 (1.2) | 12 (2.5) | 3 (0.9) | 15 (1.8) |
| Missing | 157 (72.0) | 354 (58.9) | 306 (63.2) | 205 (61.2) | 511 (62.4) |

**IPTW Poisson regression analysis using HIV cases (2014-2015)**

We identified 663 HIV cases (2014-15).

**Table S16. Inverse probability of treatment weighting (IPTW) Poisson regression models, adjusted for all demographic and socioeconomic variables, for the association between HIV incidence and Family Health Strategy (FHS) coverage, Brazil, 2007–15**

| **Variables** | **HIV incidence**  **n=3,435,068** |
| --- | --- |
|  | **RRa (CI 95%)** |
| FHS coverage |  |
| <20% | 1 |
| 100% | 0.53 (0.43-0.64) |
| Sex |  |
| Female | 1 |
| Male | 1.30 (1.10-1.53) |
| Age (years) |  |
| 13-24 | 1 |
| 25-59 | 0.76 (0.64-0.90) |
| ≥60 | 0.17 (0.10-0.27) |
| Skin color |  |
| White | 1 |
| Asian | 0.42 (0.06-2.98) |
| Pardo | 1.09 (0.91-1.31) |
| Black | 1.49 (1.12-1.99) |
| Indigenous | 1.44 (0.46-4.55) |
| Education (years of study) |  |
| >9 | 1 |
| 4-9 | 1.07 (0.87-1.33) |
| 1-4 | 0.88 (0.70-1.10) |
| No education | 0.89 (0.66-1.21) |
| Per capita expenditures - % MW |  |
| >1 | 1 |
| 0.5-1 | 1.07 (0.79-1.45) |
| 0.25-0.49 | 1.20 (0.86-1.68) |
| 0-0.24 | 1.53 (1.08-2.17) |
| Nothing declared | 1.61 (1.10-2.35) |
| Home construction material |  |
| Brick | 1 |
| Wood or taipa^1^ | 1.19 (0.96-1.47) |
| Water supply |  |
| Public network | 1 |
| Well, spring or cistern | 0.83 (0.66-1.05) |
| Lighting |  |
| Electricity | 1 |
| No electricity | 1.05 (0.79-1.39) |
| Garbage disposal |  |
| Public network | 1 |
| Burned, buried or another | 0.54 (0.41-0.70) |
| Sewage |  |
| Public network | 1 |
| Septic tank | 0.95 (0.74-1.22) |
| Rudimentary cesspit/ditch or another | 1.22 (0.99-1.49) |
| Number of individuals per room |  |
| 1 | 1 |
| 1-2 | 0.95 (0.64-1.40) |
| >2 | 0.48 (0.12-1.95) |
| Year of entry into the cohort |  |
| 2007 | 1 |
| 2008 | 1.14 (0.85-1.53) |
| 2009 | 1.20 (0.88-1.63) |
| 2010 | 1.95 (1.42-2.69) |
| 2011 | 2.24 (1.54-3.27) |
| 2012 | 2.93 (2.06-5.14) |
| 2013 | 3.44 (2.30-5.14) |
| 2014 | 7.26 (4.97-10.61) |
| 2015 | 18.74 (12.16-28.90) |
| AIDS municipal incidence among individuals in the cohort^2^ | 1.00 (1.00-1.00) |
| Time receiving Bolsa Família, months | 1.00 (1.00-1.00) |
| Municipal unemployment rate | 0.98 (0.96-1.00) |
| Hospital beds per 1,000 inhabitants | 1.05 (1.01-1.08) |

RRa=rate risk adjusted for sex, age, skin color, education, per capita household expenditures, home construction material, number of people per room, year of entry into the cohort, time receiving Bolsa Família (in months), AIDS municipal rate in the cohort, water supply, lighting, sewage, garbage disposal, municipal unemployment rate, and hospital beds per 1,000 inhabitants.

CI=Confidence Interval

**8. Complementary analysis**

**IPTW Poisson regression analysis divided in two periods (2007 to 2010 and 2011 to 2015)**

**Table S17. Inverse probability of treatment weighting (IPTW) Poisson regression models, adjusted for all demographic and socioeconomic variables, for the association between AIDS incidence and mortality and Family Health Strategy (FHS) coverage divided in two periods, Brazil, 2007–10 and 2011-15**

| **Outcome** | **2007-2010**  **n=1,860,140** | **2011-2015**  **n=1,574,882** |
| --- | --- | --- |
|  | **RRa (95%CI)** | **RRa (95%CI)** |
| AIDS incidence | 0.76 (0.67-0.85) | 0.74 (0.61-0.89) |
| AIDS mortality | 0.67 (0.54-0.84) | 0.63 (0.43-0.91) |

RRa=rate risk adjusted for sex, age, skin color, education, per capita household expenditures, home construction material, number of people per room, year of entry into the cohort, time receiving Bolsa Família (in months), AIDS municipal rate in the cohort, water supply, lighting, sewage, garbage disposal, municipal unemployment rate, and hospital beds per 1,000 inhabitants.

CI=Confidence Interval

**IPTW Poisson regression analysis from 2007 to 2010**

**AIDS incidence from 2007 to 2010**

**Table S18. Inverse probability of treatment weighting (IPTW) Poisson regression models, adjusted for all demographic and socioeconomic variables, for the association between AIDS incidence and Family Health Strategy (FHS) coverage, Brazil, 2007–10**

| **Variables** | **AIDS incidence**  **n=1,860,140** |
| --- | --- |
|  | **RRa (CI 95%)** |
| FHS coverage |  |
| <20% | 1 |
| 100% | 0.76 (0.67-0.85) |
| Sex |  |
| Female | 1 |
| Male | 0.95 (0.87-1.05) |
| Age (years) |  |
| 13-24 | 1 |
| 25-59 | 1.89 (1.69-2.10) |
| ≥60 | 0.36 (0.26-0.50) |
| Skin color |  |
| White | 1 |
| Asian | 1.38 (0.57-3.33) |
| Pardo | 1.28 (1.14-1.43) |
| Black | 1.68 (1.43-1.98) |
| Indigenous | 1.10 (0.52-2.32) |
| Education (years of study) |  |
| >9 | 1 |
| 4-9 | 1.41 (1.21-1.63) |
| 1-4 | 1.30 (1.11-1.52) |
| No education | 1.12 (0.93-1.35) |
| Per capita expenditures - % MW |  |
| >1 | 1 |
| 0.5-1 | 0.66 (0.42-1.03) |
| 0.25-0.49 | 0.65 (0.42-1.00) |
| 0-0.24 | 0.65 (0.44-0.95) |
| Nothing declared | 0.75 (0.51-1.11) |
| Home construction material |  |
| Brick | 1 |
| Wood or taipa^1^ | 1.17 (1.05-1.30) |
| Water supply |  |
| Public network | 1 |
| Well, spring or cistern | 0.89 (0.79-1.02) |
| Lighting |  |
| Electricity | 1 |
| No electricity | 1.19 (1.03-1.37) |
| Garbage disposal |  |
| Public network | 1 |
| Burned, buried or another | 0.67 (0.58-0.77) |
| Sewage |  |
| Public network | 1 |
| Septic tank | 0.79 (0.69-0.91) |
| Rudimentary cesspit/ditch or another | 0.92 (0.81-1.04) |
| Number of individuals per room |  |
| 1 | 1 |
| 1-2 | 1.03 (0.83-1.28) |
| >2 | 0.66 (0.37-1.17) |
| Year of entry into the cohort |  |
| 2007 | 1 |
| 2008 | 1.43 (1.27-1.62) |
| 2009 | 1.92 (1.68-2.20) |
| 2010 | 4.91 (4.25-5.68) |
| AIDS municipal incidence among individuals in the cohort^2^ | 1.00 (1.00-1.00) |
| Time receiving Bolsa Família, months | 1.00 (1.00-1.00) |
| Municipal unemployment rate | 1.01 (1.00-1.02) |
| Hospital beds per 1,000 inhabitants | 1.05 (1.03-1.07) |

RRa=rate risk adjusted by sex, age, skin color, education, per capita expenditures, home construction material, number of people per room, year of entry into the cohort, time receiving Bolsa Família (in months), AIDS municipal incidence in the cohort, water supply, lighting, sewage, garbage disposal, municipal unemployment rate, hospital beds per 1,000 inhabitants

1- Taipa is a construction method that consists of using clay and wood to build houses

2- Annual cumulative AIDS incidence for each individual’s municipality of residence from the study cohort

CI=Confidence Interval; FHS= Family Health Strategy; %MW - Proportional to the baseline minimum wage

**AIDS mortality from 2007 to 2010**

**Table S19. Inverse probability of treatment weighting Poisson regression models, adjusted for all demographic and socioeconomic variables, for the association between AIDS mortality and Family Health Strategy coverage, Brazil, 2007–10**

| **Variables** | **AIDS mortality**  **n=1,860,140** |
| --- | --- |
|  | **RRa (CI 95%)** |
| FHS coverage |  |
| <20% | 1 |
| 100% | 0.67 (0.54-0.84) |
| Sex |  |
| Female | 1 |
| Male | 0.97 (0.81-1.16) |
| Age (years) |  |
| 13-24 | 1 |
| 25-59 | 3.38 (2.71-4.22) |
| ≥60 | 0.82 (0.48-1.40) |
| Skin color |  |
| White | 1 |
| Asian | 1.91 (0.47-7.73) |
| Pardo | 1.38 (1.12-1.71) |
| Black | 1.75 (1.32-2.33) |
| Indigenous | 0.57 (0.08-4.06) |
| Education (years of study) |  |
| >9 | 1 |
| 4-9 | 2.74 (1.93-3.90) |
| 1-4 | 2.50 (1.75-3.58) |
| No education | 2.33 (1.55-3.50) |
| Per capita expenditures - % MW |  |
| >1 | 1 |
| 0.5-1 | 0.98 (0.34-2.79) |
| 0.25-0.49 | 0.83 (0.30-2.35) |
| 0-0.24 | 1.02 (0.38-2.69) |
| Nothing declared | 1.12 (0.42-2.95) |
| Home construction material |  |
| Brick | 1 |
| Wood or taipa^1^ | 1.10 (0.90-1.36) |
| Water supply |  |
| Public network | 1 |
| Well, spring or cistern | 0.89 (0.71-1.12) |
| Lighting |  |
| Electricity | 1 |
| No electricity | 1.24 (0.96-1.60) |
| Garbage disposal |  |
| Public network | 1 |
| Burned, buried or another | 0.69 (0.54-0.90) |
| Sewage |  |
| Public network | 1 |
| Septic tank | 0.88 (0.68-1.13) |
| Rudimentary cesspit/ditch or another | 0.95 (0.76-1.19) |
| Number of individuals per room |  |
| 1 | 1 |
| 1-2 | 1.31 (0.85-2.03) |
| >2 | 0.55 (0.17-1.76) |
| Year of entry into the cohort |  |
| 2007 | 1 |
| 2008 | 1.26 (1.01-1.57) |
| 2009 | 1.86 (1.46-2.37) |
| 2010 | 3.85 (2.94-5.05) |
| AIDS municipal incidence among individuals in the cohort^2^ | 1.00 (1.00-1.00) |
| Time receiving Bolsa Família, months | 0.99 (0.99-1.00) |
| Municipal unemployment rate | 1.01 (0.98-1.03) |
| Hospital beds per 1,000 inhabitants | 1.05 (1.02-1.09) |

RRa=rate risk adjusted by sex, age, skin color, education, per capita expenditures, home construction material, number of people per room, year of entry into the cohort, time receiving Bolsa Família (in months), AIDS municipal incidence in the cohort, water supply, lighting, sewage, garbage disposal, municipal unemployment rate, hospital beds per 1,000 inhabitants

1- Taipa is a construction method that consists of using clay and wood to build houses

2- Annual cumulative AIDS incidence for each individual’s municipality of residence from the study cohort

CI=Confidence Interval; FHS= Family Health Strategy; %MW - Proportional to the baseline minimum wage

**IPTW Poisson regression analysis from 2011 to 2015**

**AIDS incidence from 2011 to 2015**

**Table S20. Inverse probability of treatment weighting (IPTW) Poisson regression models, adjusted for all demographic and socioeconomic variables, for the association between AIDS incidence and Family Health Strategy (FHS) coverage, Brazil, 2011–15**

| **Variables** | **AIDS incidence**  **n=1,574,882** |
| --- | --- |
|  | **RRa (CI 95%)** |
| FHS coverage |  |
| <20% | 1 |
| 100% | 0.74 (0.61-0.89) |
| Sex |  |
| Female | 1 |
| Male | 1.84 (1.54-2.20) |
| Age (years) |  |
| 13-24 | 1 |
| 25-59 | 2.88 (2.21-3.75) |
| ≥60 | 0.59 (0.38-0.91) |
| Skin color |  |
| White | 1 |
| Asian | 1.64 (0.60-4.43) |
| Pardo | 1.73 (1.42-2.11) |
| Black | 1.68 (1.20-2.35) |
| Indigenous | 0.87 (0.12-6.35) |
| Education (years of study) |  |
| >9 | 1 |
| 4-9 | 1.52 (1.20-1.92) |
| 1-4 | 1.51 (1.22-1.87) |
| No education | 1.53 (1.13-2.08) |
| Per capita expenditures - % MW |  |
| >1 | 1 |
| 0.5-1 | 1.68 (1.29-2.19) |
| 0.25-0.49 | 2.15 (1.60-2.89) |
| 0-0.24 | 2.99 (2.18-4.11) |
| Nothing declared | 2.90 (1.88-4.46) |
| Home construction material |  |
| Brick | 1 |
| Wood or taipa^1^ | 1.25 (0.97-1.61) |
| Water supply |  |
| Public network | 1 |
| Well, spring or cistern | 0.89 (0.70-1.14) |
| Lighting |  |
| Electricity | 1 |
| No electricity | 1.16 (0.86-1.58) |
| Garbage disposal |  |
| Public network | 1 |
| Burned, buried or another | 0.55 (0.41-0.74) |
| Sewage |  |
| Public network | 1 |
| Septic tank | 0.85 (0.66-1.10) |
| Rudimentary cesspit/ditch or another | 0.83 (0.67-1.02) |
| Number of individuals per room |  |
| 1 | 1 |
| 1-2 | 0.65 (0.30-1.40) |
| >2 | 2.48 (1.80-7.75) |
| Year of entry into the cohort |  |
| 2011 | 1 |
| 2012 | 1.09 (0.89-1.34) |
| 2013 | 1.20 (0.94-1.53) |
| 2014 | 1.22 (0.93-1.61) |
| 2015 | 0.83 (0.49-1.39) |
| AIDS municipal incidence among individuals in the cohort^2^ | 1.00 (1.00-1.00) |
| Time receiving Bolsa Família, months | 0.99 (0.99-1.00) |
| Municipal unemployment rate | 0.98 (0.96-1.01) |
| Hospital beds per 1,000 inhabitants | 1.05 (1.01-1.09) |

RRa=rate risk adjusted by sex, age, skin color, education, per capita expenditures, home construction material, number of people per room, year of entry into the cohort, time receiving Bolsa Família (in months), AIDS municipal incidence in the cohort, water supply, lighting, sewage, garbage disposal, municipal unemployment rate, hospital beds per 1,000 inhabitants

1- Taipa is a construction method that consists of using clay and wood to build houses

2- Annual cumulative AIDS incidence for each individual’s municipality of residence from the study cohort

CI=Confidence Interval; FHS= Family Health Strategy; %MW - Proportional to the baseline minimum wage

**AIDS mortality from 2011 to 2015**

**Table S21. Inverse probability of treatment weighting (IPTW) Poisson regression models, adjusted for all demographic and socioeconomic variables, for the association between AIDS mortality and Family Health Strategy (FHS) coverage, Brazil, 2011–15**

| **Variables** | **AIDS mortality**  **n=1,574,882** |
| --- | --- |
|  | **RRa (CI 95%)** |
| FHS coverage |  |
| <20% | 1 |
| 100% | 0.63 (0.43-0.91) |
| Sex |  |
| Female | 1 |
| Male | 2.22 (1.59-3.09) |
| Age (years) |  |
| 13-24 | 1 |
| 25-59 | 5.86 (2.90-11.86) |
| ≥60 | 1.67 (0.70-4.01) |
| Skin color |  |
| White | 1 |
| Asian | 1.54 (0.21-11.20) |
| Pardo | 1.98 (1.33-2.96) |
| Black | 1.90 (1.06-3.42) |
| Indigenous | - |
| Education (years of study) |  |
| >9 | 1 |
| 4-9 | 1.49 (0.92-2.42) |
| 1-4 | 2.04 (1.36-3.06) |
| No education | 2.34 (1.34-4.06) |
| Per capita expenditures - % MW |  |
| >1 | 1 |
| 0.5-1 | 2.06 (1.23-3.44) |
| 0.25-0.49 | 2.38 (1.32-4.29) |
| 0-0.24 | 3.90 (2.11-7.20) |
| Nothing declared | 3.46 (1.55-7.74) |
| Home construction material |  |
| Brick | 1 |
| Wood or taipa^1^ | 1.02 (0.58-1.79) |
| Water supply |  |
| Public network | 1 |
| Well, spring or cistern | 0.66 (0.40-1.11) |
| Lighting |  |
| Electricity | 1 |
| No electricity | 0.68 (0.31-1.49) |
| Garbage disposal |  |
| Public network | 1 |
| Burned, buried or another | 0.71 (0.40-1.25) |
| Sewage |  |
| Public network | 1 |
| Septic tank | 0.62 (0.37-1.03) |
| Rudimentary cesspit/ditch or another | 0.73 (0.49-1.07) |
| Number of individuals per room |  |
| 1 | 1 |
| 1-2 | 0.70 (0.17-2.89) |
| >2 | 3.69 (0.53-25.83) |
| Year of entry into the cohort |  |
| 2011 | 1 |
| 2012 | 1.05 (0.73-1.50) |
| 2013 | 1.02 (0.62-1.66) |
| 2014 | 0.96 (0.55-1.66) |
| 2015 | 0.16 (0.02-1.17) |
| AIDS municipal incidence among individuals in the cohort^2^ | 1.00 (1.00-1.00) |
| Time receiving Bolsa Família, months | 0.99 (0.99-1.00) |
| Municipal unemployment rate | 1.01 (0.97-1.06) |
| Hospital beds per 1,000 inhabitants | 1.09 (1.04-1.15) |

RRa=rate risk adjusted by sex, age, skin color, education, per capita expenditures, home construction material, number of people per room, year of entry into the cohort, time receiving Bolsa Família (in months), AIDS municipal incidence in the cohort, water supply, lighting, sewage, garbage disposal, municipal unemployment rate, hospital beds per 1,000 inhabitants

1- Taipa is a construction method that consists of using clay and wood to build houses

2- Annual cumulative AIDS incidence for each individual’s municipality of residence from the study cohort

CI=Confidence Interval; FHS= Family Health Strategy; %MW - Proportional to the baseline minimum wage

**9. Triangulation analysis**

**Table S22. Triangulation analysis for AIDS incidence and mortality rates, Brazil, 2007-15**

| **Rates** | **Poisson IPTW regression** | **Cox regression** |
| --- | --- | --- |
|  | **RRa (CI 95%)** | **HRa (CI 95%)** |
| AIDS Incidence | 0.76 (0.68-0.84) | 0.76 (0.68-0.84) |
| AIDS Mortality | 0.68 (0.56-0.82) | 0.68 (0.56-0.82) |

RRa=rate risk adjusted by sex, age, skin color, education, per capita household expenditures, home construction material, number of people per room, year of entry into the cohort, time receiving Bolsa Família (in months), AIDS municipal incidence in the cohort, water supply, lighting, sewage, garbage disposal, municipal unemployment rate, hospital beds per 1,000 inhabitants

HRa= hazard ratio adjusted by sex, age, skin color, education, per capita household expenditures, home construction material, number of people per room, year of entry into the cohort, time receiving Bolsa Família (in months), AIDS municipal incidence in the cohort, water supply, lighting, sewage, garbage disposal, municipal unemployment rate, hospital beds per 1,000 inhabitants

CI=Confidence Interval
