# Supplementary figures and images for: The impact of primary health care on AIDS incidence and mortality: A cohort study of 3.4 million Brazilians
Source: PLoS Med. 2024 Jul 11;21(7):e1004302. doi: 10.1371/journal.pmed.1004302 (PMC11272382; doi:10.1371/journal.pmed.1004302)

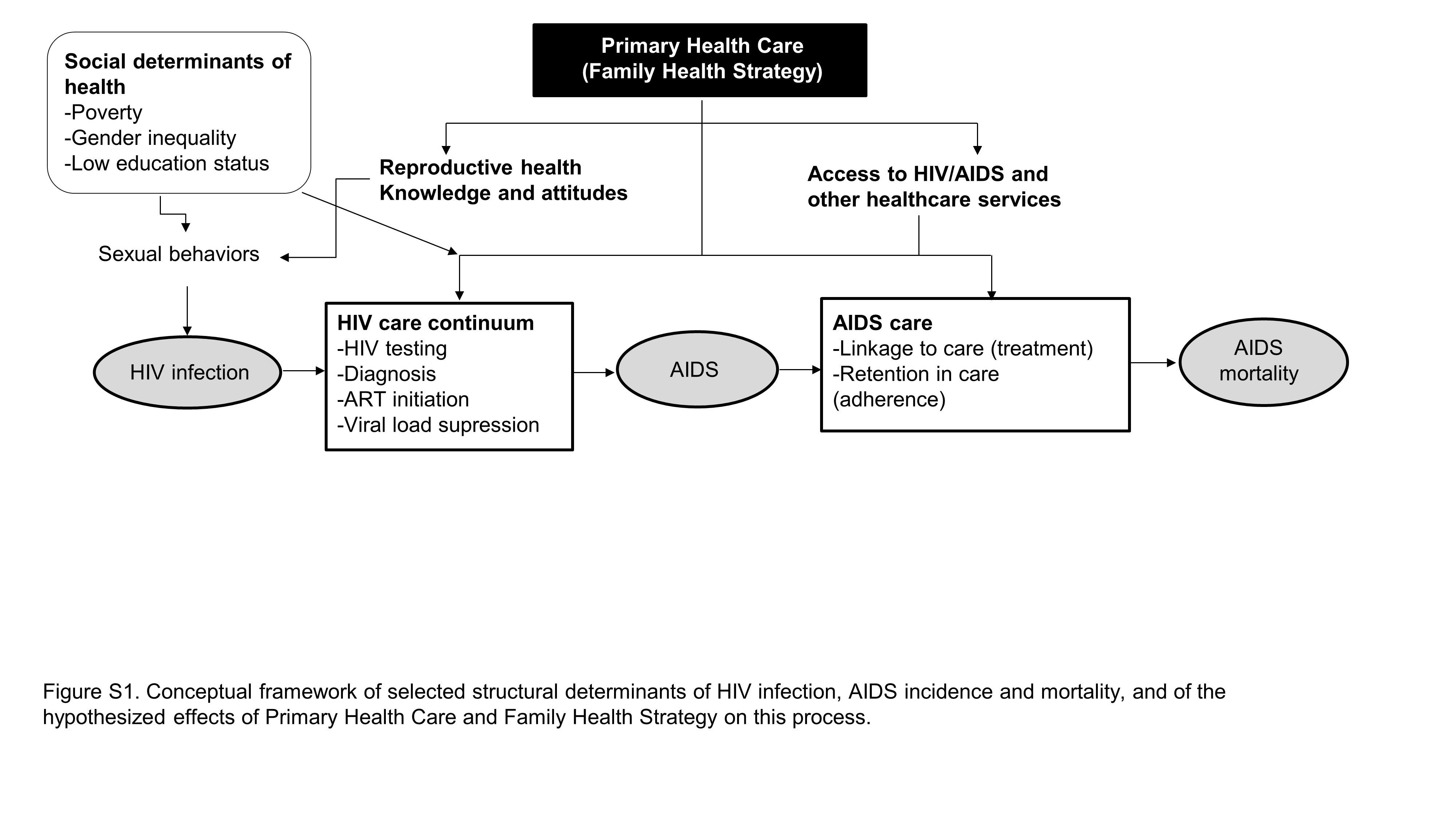

Supplement: S1 Fig — (TIF) [file pmed.1004302.s003.tif]
